# Disruption of the Clock Component BMAL1 in HDM-induced Asthma Causes GC Resistance in Airway Epithelial Cells by Regulating GR Phosphorylation through the DUSP4-p38MAPK Pathway

**DOI:** 10.7150/ijbs.119486

**Published:** 2025-10-10

**Authors:** Haohua Huang, Hua Liao, Yixin Chen, Minxuan Hu, Xiaoxiao Jiang, Qi Yu, Yimei Gao, Huimin Yang, Jinzhong Zhuo, Dongyu Liu, Liping Huang, Jinming Zhang, Yanqun Li, Yuhan Du, Xiaodong Lin, Xiaojing Meng, Fei Zou, Mengchen Zou, Jieyu Wu, Shaoxi Cai, Hangming Dong

**Affiliations:** 1 Chronic Airways Diseases Laboratory, Department of Respiratory and Critical Care Medicine, Nanfang Hospital, Southern Medical University, Guangzhou, China.; 2 Department of Respiratory and Critical Care Medicine, The Fifth Affiliated Hospital of Southern Medical University, Guangzhou, China.; 3 Department of Pathology, The First Affiliated Hospital of Guangzhou Medical University, Guangzhou, China.; 4 School of Public Health, Southern Medical University, Guangzhou, China.; 5 Department of Endocrinology, Nanfang Hospital, Southern Medical University, Guangzhou, China.

**Keywords:** BMAL1, Circadian rhythm, Asthma, Glucocorticoid Resistance, Glucocorticoid Receptor, p38MAPK

## Abstract

Circadian rhythm disruption has been increasingly implicated in asthma and glucocorticoid (GC) resistance. In this study, we discovered that disruption of the brain and muscle ARNT-like 1 (BMAL1), a significant activator of the circadian clock transcription, not only exacerbated allergic inflammation but also induced GC resistance. The absence of BMAL1 intensified airway inflammation by activating the NF-κB and AP-1 pathways and also impaired the anti-inflammatory effect of GC. Our findings indicated that the deletion of *BMAL1* reduced the phosphorylation level of the GC receptor (GR-Ser211), which has a direct effect on the efficacy of GC and serves as a key indicator of GR activation. Additionally, BMAL1 has a negative regulatory effect on the phosphatase dual specificity protein phosphatase 4 (DUSP4) of p38 mitogen-activated protein kinase (p38MAPK), which plays a crucial role in the phosphorylation of GR. Consequently, our findings suggest that the absence of BMAL1 results in the resistance of airway epithelial cells to GC due to the inhibition of GR phosphorylation via the DUSP4-p38MAPK axis in HDM-induced asthma. We demonstrated that the inhibition of DUSP4 restored GR activation and improved GC responsiveness, highlighting a potential therapeutic strategy for GC resistance driven by circadian disruption. Regulating the sleep disorder and circadian rhythm of patients with asthma could become a potential treatment to increase GC sensitivity.

## Introduction

Asthma is a common chronic inflammatory disorder of the airways, affecting more than 300 million individuals worldwide and showing an increasing global prevalence 1, 2. Glucocorticoids (GCs) remain the mainstay of asthma management. However, a substantial subset of patients exhibits poor responsiveness to GC therapy, commonly referred to as steroid-resistant asthma (SRA) 3-6. SRA significantly compromises disease control, worsens prognosis, and places a heavy burden on healthcare systems 7, 8. While multiple mechanisms have been proposed, including glucocorticoid receptor (GR) dysfunction, the molecular basis underlying GC resistance remains poorly understood.

The airway epithelium, as the frontline barrier and immune modulator, plays a critical role in asthma pathogenesis 9-12, and increasing evidence indicates that its dysfunction may contribute to GC resistance in asthma 13-15. Glucocorticoids function through binding to cytoplasmic GRs to modulate anti-inflammatory gene transcription, and reduced glucocorticoid efficacy has been linked to impaired GR phosphorylation, particularly at Ser211 16-21. Among environmental triggers, house dust mite (HDM) is a major perennial allergen in asthma 22. Although the downstream alterations in GR signaling have been partially characterized, the upstream regulatory mechanisms, especially in the context of HDM-induced asthma, remain poorly defined.

The circadian clock is a fundamental regulator of physiological processes, including immune responses, metabolism, and hormone signaling 22, 23. Clinical observations show that asthma symptoms worsen at night and the early morning. Growing evidence have revealed a correlation between asthma patients with disturbed circadian rhythms and increased difficulty in managing their symptoms, reflecting underlying circadian influences 24-26. Genetic studies further support this connection: mice with circadian clock gene deletion exhibit more severe airway inflammation and reduced GC sensitivity 27, 28. The brain and muscle ARNT-like 1 (BMAL1) and the circadian locomotor output cycle protein kaput (CLOCK) are the two major transcriptional regulators of the circadian system 29. Mechanistically, the CLOCK/BMAL1 complex has been reported to regulate GR function, suggesting a mechanistic link between the circadian clock and GR signaling 30, 31.

Based on these findings, we hypothesized that *BMAL1* deletion disrupts GR signaling and promotes GC resistance in asthma. To test this, we deleted *BMAL1* in airway epithelial cells and established a mouse model to examine how its loss alters GC responsiveness in HDM-induced asthma.

We found that circadian disruption and *BMAL1* deficiency aggravated HDM-induced airway inflammation and diminished the anti-inflammatory efficacy of dexamethasone (DEX). BMAL1 loss leading to dual specificity protein phosphatase 4 (DUSP4) upregulation, reduced p38 mitogen-activated protein kinase (p38MAPK) activation, and decreased GR-Ser211 phosphorylation, thereby impairing GC responsiveness. Importantly, DUSP4 inhibition restored p38MAPK activity and GR function, highlighting the DUSP4-p38MAPK-GR axis as a potential therapeutic target for circadian-related GC resistance in asthma.

## Results

### Disruption of the circadian clock worsens asthma symptoms in patients

To investigate the association between circadian rhythms and asthma severity, we collected clinical data from asthma patients who received standardized treatment according to the GINA 2023 guideline at the Department of Respiratory and Critical Care Medicine, Nanfang Hospital, Southern Medical University between September 2023 and September 2024.

All patients voluntarily completed the Morningness Eveningness Questionnaire (MEQ-SA), which is used to assess the chronotype and sleep quality of patients. The Asthma Control Test (ACT) questionnaire was used to measure the level of asthma control. A total of 70 patients with asthma were included in the study, with 34 early chronotypes and 36 late chronotypes. The distributions of demographics (age, gender) were as follows: early chronotype (mean ± SD age, 42.9 ± 15.0; 64.7% female) and late chronotype (mean ± SD age, 44.1 ± 12.5; 66.7% female). Late chronotype patients had higher rates of uncontrolled asthma compared with early chronotype patients (41.7% vs 17.6%). Moreover, late chronotype patients more frequently experienced ≥ 2 annual exacerbations (16.7% vs 5.88%) and ≥ 3 exacerbations (11.1% vs 0%). The late phenotype exhibited increased symptom severity and required a higher average daily hormone dosage (466.7µg/d vs 422.4µg/d) (Table 1).

These findings suggest that disrupted circadian alignment may be associated with worsened asthma control and increased dependence on GC therapy.

### Dysregulated BMAL1 in airway epithelial cells is correlated with severe asthma

**Figure 1:**
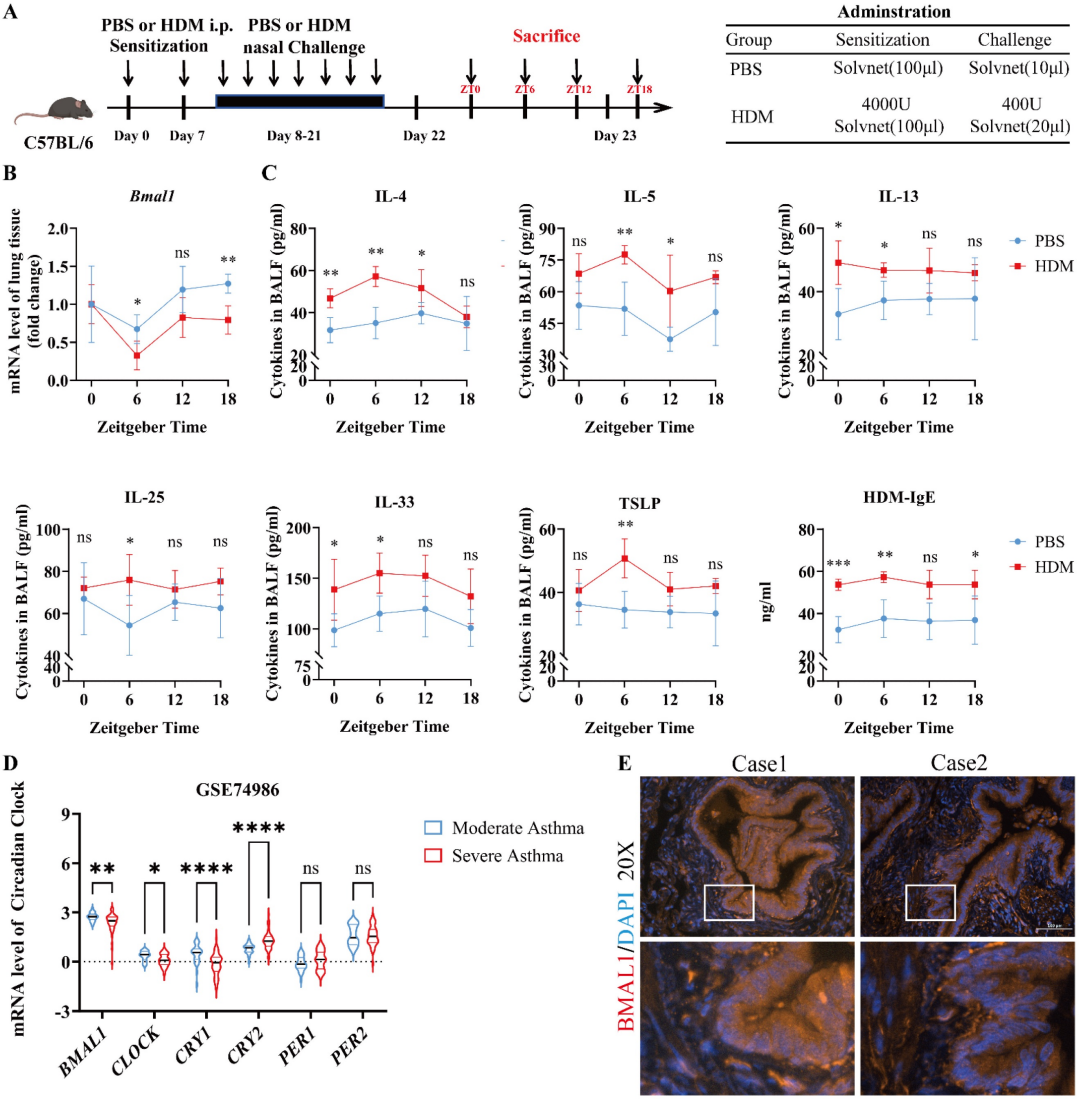
** Dysregulated BMAL1 in airway epithelial cells is correlated with severe asthma. (A)** Schematic diagram of the experimental protocol for the modeling and timing of sample collection in HDM experimental asthmatic mice (ZT0, ZT6, ZT12, and ZT18). **(B)** The mRNA level of *Bmal1* in mouse lung tissues at 4 time points (ZT0, ZT6, ZT12, and ZT18) throughout the day. **(C)** The levels of IL-4, IL-5, IL-13, IL-25, IL-33 and TSLP in BALF and HDM-IgE in serum at 4 time points were measured via ELISA (ZT0, ZT6, ZT12, and ZT18). **(D)** mRNA expression levels of *BMAL1*, *CLOCK*, *CRY1*, *CRY2*, *PER1* and *PER2* in bronchial exfoliating cells from human samples in the GSE74986 dataset.** (E)** Immunofluorescence staining demonstrated that BMAL1 is expressed mainly in bronchial epithelial cells from human lung samples (scale bar =100 μm). *P<0.05, **P<0.01, ***P<0.001, ****P<0.0001

To investigate how circadian disruption affects asthma progression, we established a sleep deprivation (SD) model of HDM-induced asthma in C57BL/6J mice (Fig S1A). While SD does not entirely replicate central clock dysfunction, it functions as a surrogate for misaligned sleep-wake cycles. Compared with HDM-treated mice, SD-HDM mice exhibited more severe airway inflammation (Fig S1B). We subsequently investigated the expression of rhythmic mRNAs in the lungs of SD-HDM mice (Fig S1C) and found a significant downregulation of *Bmal1.* Moreover, the immunofluorescence revealed that BMAL1 is prominently expressed within the airway epithelium (Fig S1D-E). To investigate the influence of circadian rhythm on allergic inflammation, we analyzed the oscillating expression pattern of *Bmal1* in lung tissues from PBS-treated and HDM-treated mice (Fig 1A). A decrease in the rhythmic oscillation of *Bmal1* was observed in HDM mice (Fig 1B). We also observed significant variability in inflammatory responses in the bronchoalveolar lavage fluid (BALF) of HDM mice, particularly an increase in T1 inflammatory factors (IL-6 and IFN-γ) (Fig S1F) and T2 inflammatory markers (IL-4, IL-5, IL-13, IL-25, IL-33, TSLP and IgE) during the low peak of *Bmal1* (Fig 1C). To further examine the role of circadian disorders in airway epithelial cells, we conducted a comprehensive analysis of transcriptomic datasets from bronchial exfoliating cells (GSE74986). Our analysis revealed that individuals diagnosed with severe asthma presented markedly lower BMAL1 levels than individuals with moderate asthma did (Fig 1D). As we expected, HDM stimulation similarly reduced BMAL1 expression in HBECs (Fig S1G). Interestingly, the RNA sequencing results suggested that *CLOCK* and Cryptochrome1 (*CRY1*) followed the same trend as *BMAL1*. Other circadian rhythm components, including PERIOD1 (*PER1*) and PERIOD2 (*PER2*), did not differ between the severe asthma group and the moderate asthma group (Fig 1D). Furthermore, immunofluorescence of human lung tissue revealed BMAL1 expression in the central region of airway epithelial cells (Fig 1E).

These findings demonstrate that allergic stimuli suppress BMAL1 expression in airway epithelial cells, and that BMAL1 dysregulation correlates with heightened inflammation and asthma severity. Together, this suggests a significant association between asthma progression and BMAL1 dysfunction in the airway epithelium.

### BMAL1 deficiency in HBECs increased the HDM-induced inflammatory response

**Figure 2:**
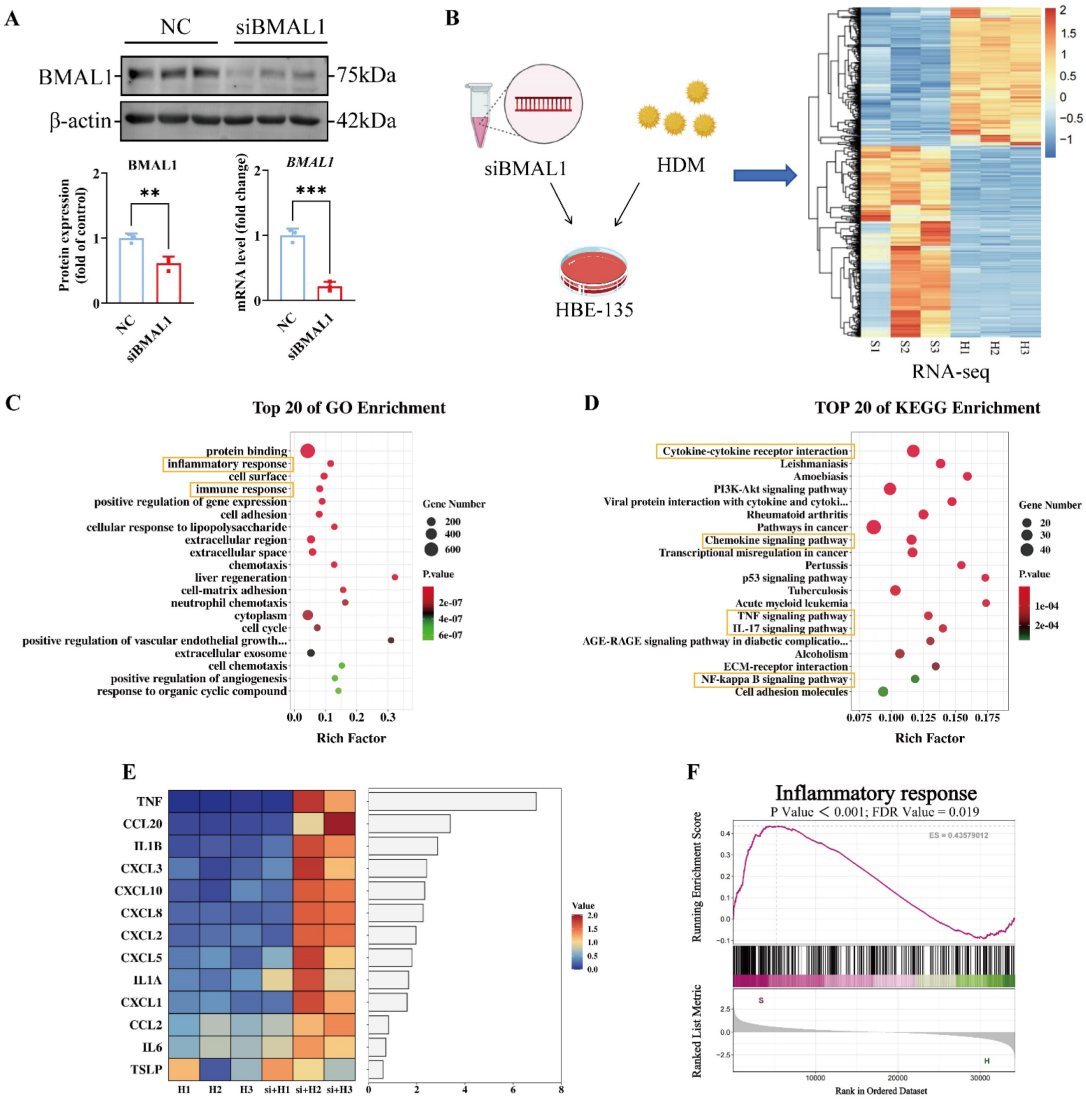
** BMAL1 deficiency in HBECs increased the HDM-induced inflammatory response. (A)** Western blotting and qPCR showing the knockdown efficiency of BMAL1 siRNA. **(B)** RNA sequencing of HBECs after BMAL1 depletion for 24 h. **(C-D)** Pathway enrichment analysis showing DEGs related to different pathway terms. **(E)** Heatmap showing the targeted cytokine and chemokine related components. **(F)** GSEA showing the increase in the inflammatory response in the BMAL1-silenced background. *P<0.05, **P<0.01, ***P<0.001, ****P<0.0001

We subsequently investigated the potential mechanisms by which BMAL1 deficiency in airway epithelial cells contributes to the HDM-induced inflammatory response. We knocked down *BMAL1* via a small interfering RNA (siRNA) (Fig 2A) and subsequently performed RNA sequencing (Fig 2B). Principal component analysis revealed clear separation between the siBMAL1-treated group and the HDM-treated group (Fig S2A-B). The BMAL1 deficiency profoundly altered the HBECs transcriptome, with 657 significantly upregulated genes and 486 significantly downregulated genes (Fig S2C). And the sequencing results revealed *BMAL1* downregulation and revealed marked alterations in circadian clock-related genes in the siBMAL1 group (Fig S2D-E).

Gene ontology (GO) enrichment revealed that the HDM-induced inflammatory response was significantly enhanced in the BMAL1 knockdown cells (Fig 2C). In the BMAL1-knockdown cells, the Kyoto Encyclopedia of Genes and Genomes (KEGG) enrichment suggested upregulation of inflammation-related pathways including TNF, IL-17 and NF-κB pathways, involving multiple cytokines and chemokines (Fig 2D). Furthermore, a heatmap of cytokines and chemokines revealed an increase in the expression of inflammatory markers in BMAL1-knockdown cells (Fig 2E). Consistently, gene set enrichment analysis (GSEA) suggested increased inflammation after BMAL1 knockdown (Fig 2F).

These results indicate that BMAL1 functions as a negative regulator of HDM-induced inflammatory responses in airway epithelial cells.

### HDM induced more severe allergic inflammation in *Bmal1*^-/-^ mice

**Figure 3:**
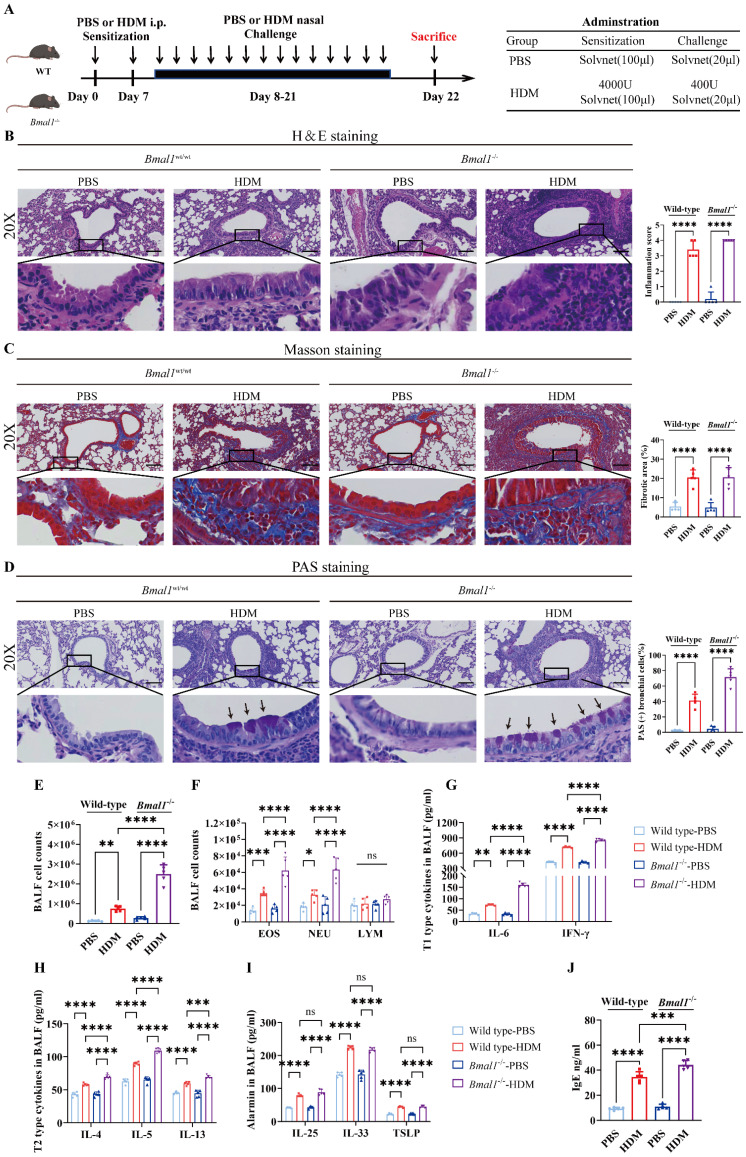
** HDM induced more severe allergic inflammation in *Bmal1*^-/-^ mice. (A)** Schematic diagram of the experimental protocol for HDM sensitization and challenge. **(B)** Representative images of H&E-stained lung tissue sections from the different groups (scale bar =100 μm). The inflammation score was determined. **(C-D)** The amount of collagen around the airways and the percentages of PAS-positive airway epithelial cells were quantified (scale bar =100 μm). **(E)** Numbers of total cells in the BALF. **(F)** Examination of BALF samples showing the number of differential inflammatory cells. **(G-I)** ELISA results showing the levels of IL-6, IFN-γ, IL-4, IL-5, IL-13, IL-25, IL-33 and TSLP in the BALF. **(J)** Total serum IgE levels were assessed via ELISA. *P<0.05, **P<0.01, ***P<0.001, ****P<0.0001, n = 5.

To assess the role of BMAL1 in allergic airway inflammation, we established an HDM-induced asthma model using wild-type (WT) mice and *Bmal1*^-/-^ mice (Fig 3A). The H&E, Masson and PAS staining showed that the HDM challenge induced severe airway inflammatory infiltration, mucus secretion and airway remodeling in *Bmal1*^-/-^ mice (Fig 3B-D). BALF of *Bmal1*^-/-^ mice demonstrated a pronounced increase in inflammatory cells, including eosinophils (EOSs) and neutrophils (NEUs), after HDM challenge (Fig 3E-F). The levels of T1 cytokines, including IFN-γ and IL-6, were markedly elevated in the knockout mice (Fig 3G). Moreover, T2 cytokines (IL-4, IL-5, and IL-13) were also elevated. Serum IgE and BALF alarmin levels were significantly elevated in *Bmal1*^-/-^ mice following HDM challenge (Fig 3H-J). Together, these results indicate that *Bmal1* deletion leads to more severe airway inflammation upon HDM challenge.

### BMAL1 deficiency impairs GC responsiveness in HBECs

**Figure 4:**
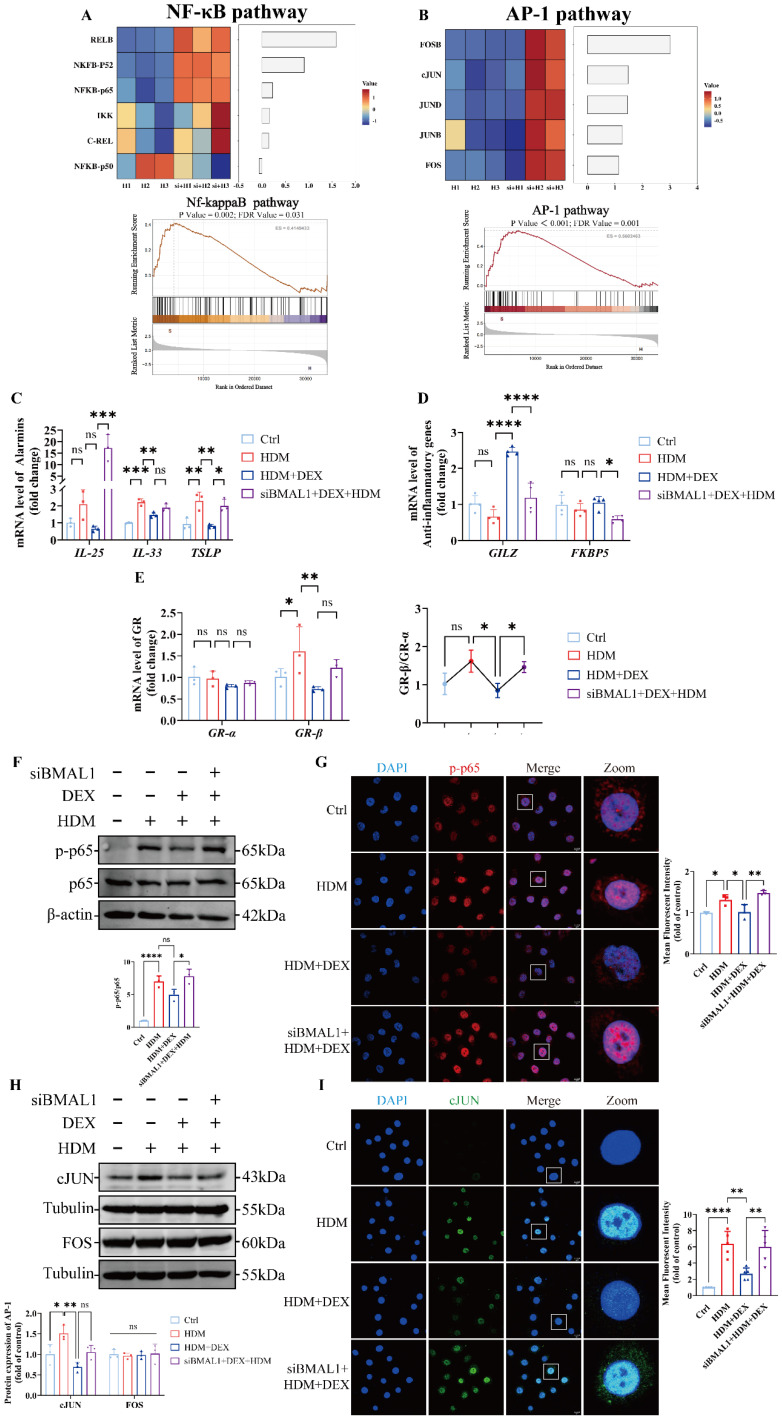
** BMAL1 deficiency impaired GC responsiveness in HBECs. (A-B)** RNA** s**equencing data showing the expression of the NF-κB pathway and AP-1 pathway. **(C-D)** mRNA levels of IL-25, IL-33, TSLP, GILZ and FKBP5 in HBECs. **(E)** The mRNA levels of GRα and GRβ and the ratios in the HBECs. **(F-G)** The protein expression of p-p65 and p65 in HBECs was measured by western blotting and immunofluorescence staining (scale bar =10 μm). **(H)** The protein expression of cJUN and FOS in HBECs was determined by western blotting. **(I)** Immunofluorescence showing the expression of cJUN in HBECs (scale bar =10 μm). *P<0.05, **P<0.01, ***P<0.001, ****P<0.0001

Our previous data showed that *Bmal1*^-/-^ mice exhibit a mixed granulocytic asthma phenotype, featuring EOS and NEU airway inflammation, and demonstrated resistance to GC treatment 32, 33. Given the known role of NF-κB and AP-1 as GC-responsive transcription factors, we examined their transcriptional activity in BMAL1-deficient cells 34, 35. RNA-seq analysis revealed that the transcription levels of NF-κB and AP-1 pathways were significantly upregulated in BMAL1-knockdown cells (Fig 4A-B).

To determine whether BMAL1 influences GC responsiveness, we treated BMAL1-knockdown HBECs with DEX. We found that BMAL1 deficiency markedly diminished the anti-inflammatory effect of DEX on IL-25 and TSLP (Fig 4C). These findings suggest that BMAL1 loss compromises the anti-inflammatory efficacy of GC treatment.

We further assessed GR transcriptional activity by quantifying the expression levels of two well-established GC-responsive genes, glucocorticoid-induced leucine zipper (GILZ) and FK506 binding protein 5 (FKBP5). Both genes were markedly downregulated in BMAL1-deficient cells (Fig 4D). In addition, the ratio of GR-β to GR-α expression was significantly increased in BMAL1-knockdown cells, suggesting an inhibitory shift in GR isoform balance and reduced GC sensitivity (Fig 4E) 36, 37.

Consistent with transcriptomic findings, immunoblotting confirmed elevated protein levels of phosphorylated NF-κB p65 (p-p65) and cJUN in BMAL1-deficient HBECs (Fig 4F-I), supporting a role for BMAL1 in suppressing pro-inflammatory signaling and maintaining epithelial GC responsiveness.

### BMAL1 deletion inhibited the p38MAPK pathway and reduced GR-Ser211 phosphorylation in HBECs

**Figure 5:**
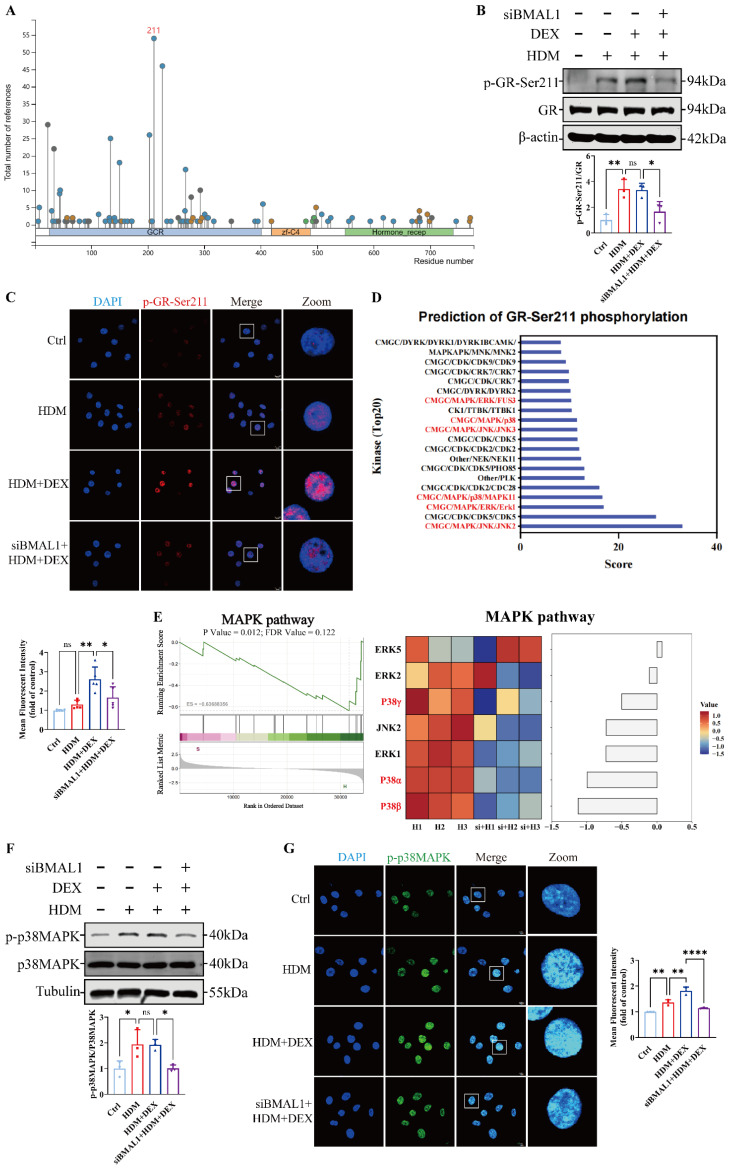
** BMAL1 deletion inhibited the p38MAPK pathway and reduced GR-Ser211 phosphorylation in HBECs. (A)** PhosphoSitePlus prediction suggested the phosphorylation site of GR. **(B-C)** Western blotting and immunofluorescence revealed the phosphorylation of GR-Ser211 in HBECs (scale bar =10 μm). **(D)** GPS6.0 predicted the potential kinases that affect GR-Ser211 phosphorylation. **(E)** Sequencing data revealed downregulation of the MAPK pathway. **(F-G)** The protein expression of p-p38MAPK in HBECs was measured by western blotting and immunofluorescence (scale bar =10 μm). *P<0.05, **P<0.01, ****P<0.0001

To determine whether BMAL1 influences GR activation, we first assessed GR mRNA levels using RNA-seq, which revealed no significant changes following BMAL1 knockdown (Fig S2F). Given that phosphorylation is a key marker of GR activation 34, 35, 38, 39, we used the PhosphoSitePlus® database to identify Ser211 as the predominant site of GR phosphorylation, consistent with previous studies (Fig 5A) 40, 41. We found that the phosphorylation of GR at Ser211 increased coincided with the increasing duration of DEX treatment (Fig S3A). However, subsequent western blot and immunofluorescence staining revealed that phosphorylation at GR-Ser211 was markedly diminished after BMAL1 knockdown (Fig 5B-C), suggesting that BMAL1 is required for GR activation.

To identify the upstream signaling events mediating this effect, we utilized the kinase prediction tool GPS 6.0 to screen for candidate kinases targeting GR-Ser211 (Fig 5D).

Integrating these predictions with our transcriptomic data, we hypothesized that BMAL1 may enhance GR phosphorylation through regulation of the MAPK pathway (Fig 5E). Among the three classical MAPK branches, p38MAPK, extracellular regulated protein kinase (ERK) and cJUN N-terminal kinase (JNK) 42, only p38MAPK phosphorylation was significantly diminished in BMAL1-knockdown cells, whereas ERK and JNK activation remained unchanged (Fig 5F-G; Fig S3B).

To validate MAPK activity changes, we applied the MAPK Pathway Activity Score (MPAS), a gene expression-based metric reflecting MAPK pathway output 43. Upon BMAL1 knockdown, MPAS and downstream transcriptional analyses suggested that p38MAPK was repressed, whereas the ERK pathway was activated (Fig S3B, Fig S4A-B). These findings indicate that BMAL1 supports GR activation by maintaining p38MAPK signaling, and its loss results in impaired GR phosphorylation and diminished GC responsiveness.

### *Bmal1*^-/-^ mice display GC resistance in the HDM-induced asthma model

**Figure 6:**
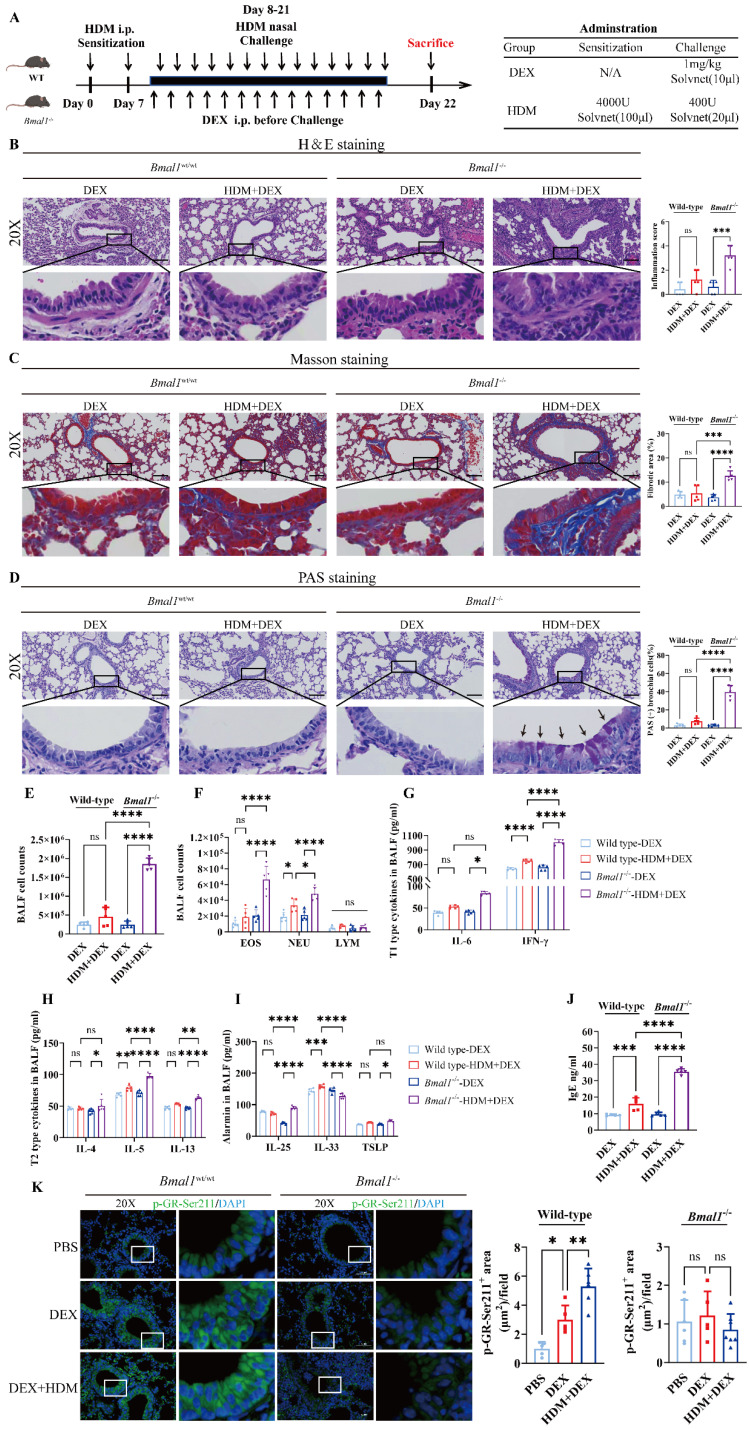
***Bmal1*^-/-^ mice display GC resistance in the HDM-induced asthma model. (A)** Schematic diagram of the experimental protocol for HDM sensitization, followed by HDM challenge and DEX treatment. **(B)** Representative images of H&E-stained lung tissue sections from the different groups. (Scale bar = 100 μm). The inflammation score was determined. **(C-D)** The amount of collagen around the airways and the percentages of PAS-positive airway epithelial cells were quantified (scale bar = 100 μm). **(E)** Numbers of total cells in the BALF. **(F)** Examination of BALF samples showing the number of differential inflammatory cells. **(G-I)** ELISA results showing the levels of IL-6, IFN-γ, IL-4, IL-5, IL-13, IL-25, IL-33 and TSLP in the BALF. **(J)** Total serum IgE levels were assessed via ELISA.** (K)** Immunofluorescence staining demonstrated the phosphorylation of GR-Ser211 in airway epithelial cells (scale bar =50 μm). *P<0.05, **P<0.01, ***P<0.001, ****P<0.0001, n = 5.

Next, we assessed the efficacy of GC treatment following BMAL1 deletion *in vivo* (Fig 6A). Histological examination revealed that DEX markedly alleviated airway inflammation, mucus secretion, and structural remodeling in WT mice. However, these anti-inflammatory effects were substantially diminished in *Bmal1*^-/-^ mice (Fig 6B-D).

In addition, the classification of cells in the BALF revealed that DEX failed to ameliorate EOS and NEU inflammation in *Bmal1*^-/-^ mice (Fig 6E-F). The levels of inflammatory factors, including T1 and T2 cytokines, were not significantly reduced in the BALF of *Bmal1*^-/-^ mice after DEX treatment in contrast to the robust suppression observed in WT controls (Fig 6G-J). Immunofluorescence staining revealed a significant decrease in GR-Ser211 phosphorylation in airway epithelial cells from *Bmal1*^-/-^ mice (Fig 6K). Collectively, these findings demonstrate BMAL1 is essential for the anti-inflammatory effects of GC therapy in asthma by sustaining GR phosphorylation.

### BMAL1 repressed DUSP4 transcription to regulate p38MAPK signaling

**Figure 7:**
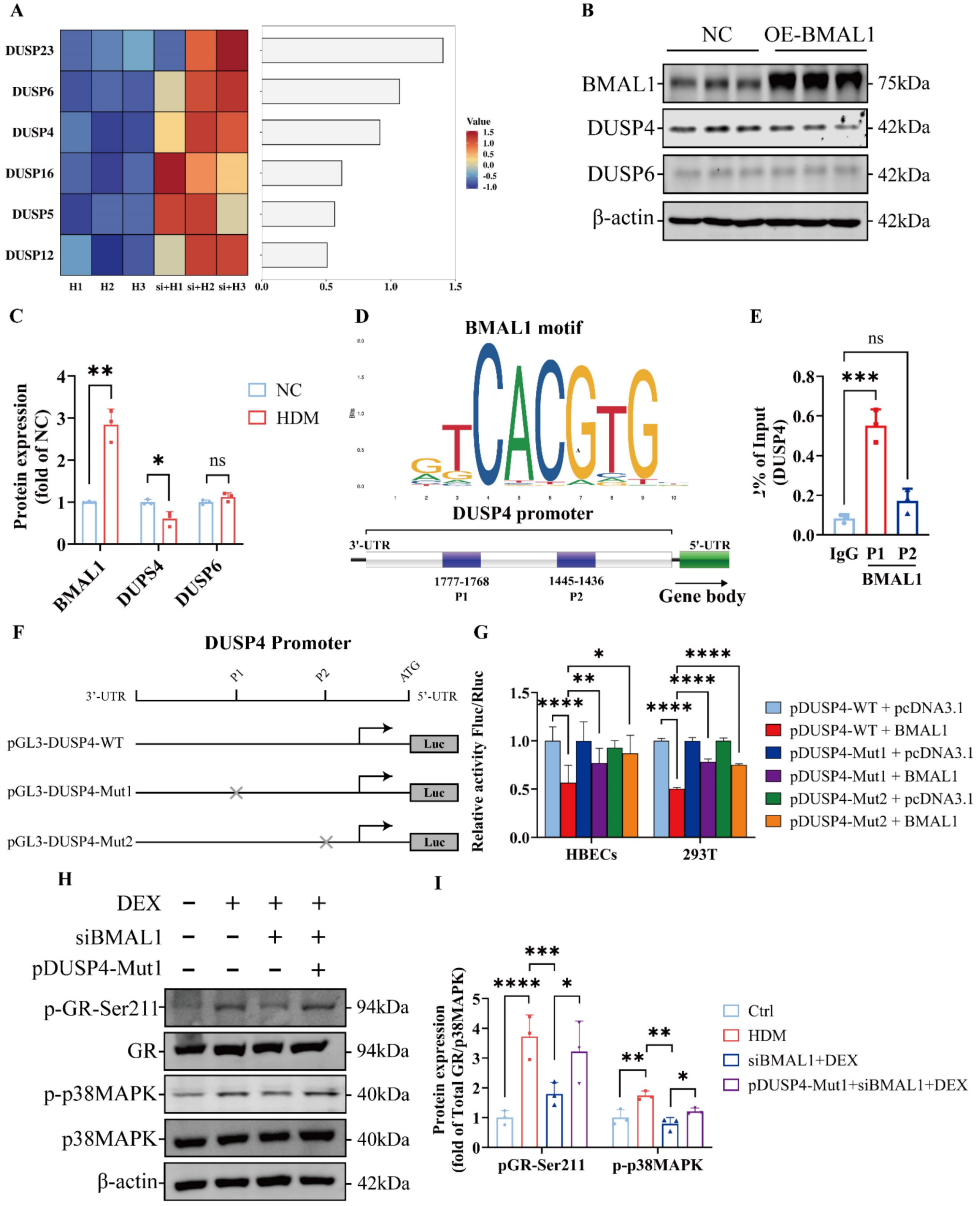
** BMAL1 repressed DUSP4 transcription to regulate p38MAPK signaling. (A)** Sequencing data demonstrated the downregulation of DUSP family members. **(B-C)** The protein expression and quantification of BMAL1, DUSP4 and DUSP6 in HBECs after BMAL1 overexpression. **(D)** Schematic representation of the DUSP4 promoter with the indicated regions. **(E)** ChIP analysis of the DUSP4 promoter in HBECs. Immunoprecipitation was performed using anti-BMAL1 and IgG antibodies.** (F)** Schematic representation of the transfection of DUSP4 plasmids and the luciferase reporter assay. **(G)** Dual-luciferase reporter activity was measured and normalized against Renilla luciferase activity in the HBECs and 293T that were transfected with the pGL3-DUSP4-WT, pGL3-DUSP4-Mut1 or pGL3-DUSP4-Mut2 plasmid. **(H-I)** Protein expression and quantification of p-GR-Ser211 and p-p38MAPK in HBECs after transfection with siBMAL1 or pGL3-DUSP4-Mut1.

To explore how BMAL1 modulates the p38MAPK pathway, we examined its regulation of dual-specificity phosphatases (DUSPs), which serve as negative regulators of MAPK activity 44. The DUSP4 and DUSP6 have been shown to play key roles in the modulating MAPK pathways 45. We found that the transcription levels of DUSPs were significantly increased in BMAL1-knockdown cells (Fig 7A). To further study the transcriptional regulation of DUSPs by BMAL1, we established a BMAL1-overexpressed HBEC line via plasmid transfection. We found that only DUSP4 was significantly downregulated after BMAL1 overexpression (Fig S5).

Western blot results further confirmed that BMAL1 negatively regulates DUSP4 expression (Fig 7B-C).

To test whether DUSP4 is a direct transcriptional target of BMAL1, we analyzed the promoter region of the DUSP4 gene using JASPAR and the NCBI promoter database, identifying two putative BMAL1-binding motifs (P1: -1768 to -1777; P2: -1436 to -1445) (Fig 7D). Chromatin immunoprecipitation (ChIP) assays confirmed strong BMAL1 enrichment at the P1 site, with modest, non-significant enrichment at the P2 site (Fig 7E).

We further constructed the pGL3-DUSP4-WT, pGL3-DUSP4-Mut1 and pGL3-DUSP4-Mut2 plasmids for the luciferase reporter assay (Fig 7F). The results revealed that BMAL1 overexpression significantly reduced DUSP4 promoter activity in luciferase reporter assays. Mutation of the P1 or P2 binding site partially restored DUSP4 expression, indicating these motifs mediate BMAL1-dependent repression (Fig 7G). Moreover, the western blotting results also suggested that a mutation in the P1 region of DUSP4 reversed the repressive effect of BMAL1 on the p38MAPK pathway (Fig 7H-I).

Collectively, these findings demonstrate that BMAL1 directly binds to the DUSP4 promoter and represses its transcription, thereby sustaining p38MAPK activity and downstream GR phosphorylation.

### Inhibition of DUSP4 restored GC sensitivity in BMAL1-deficient airway epithelial cells

**Figure 8:**
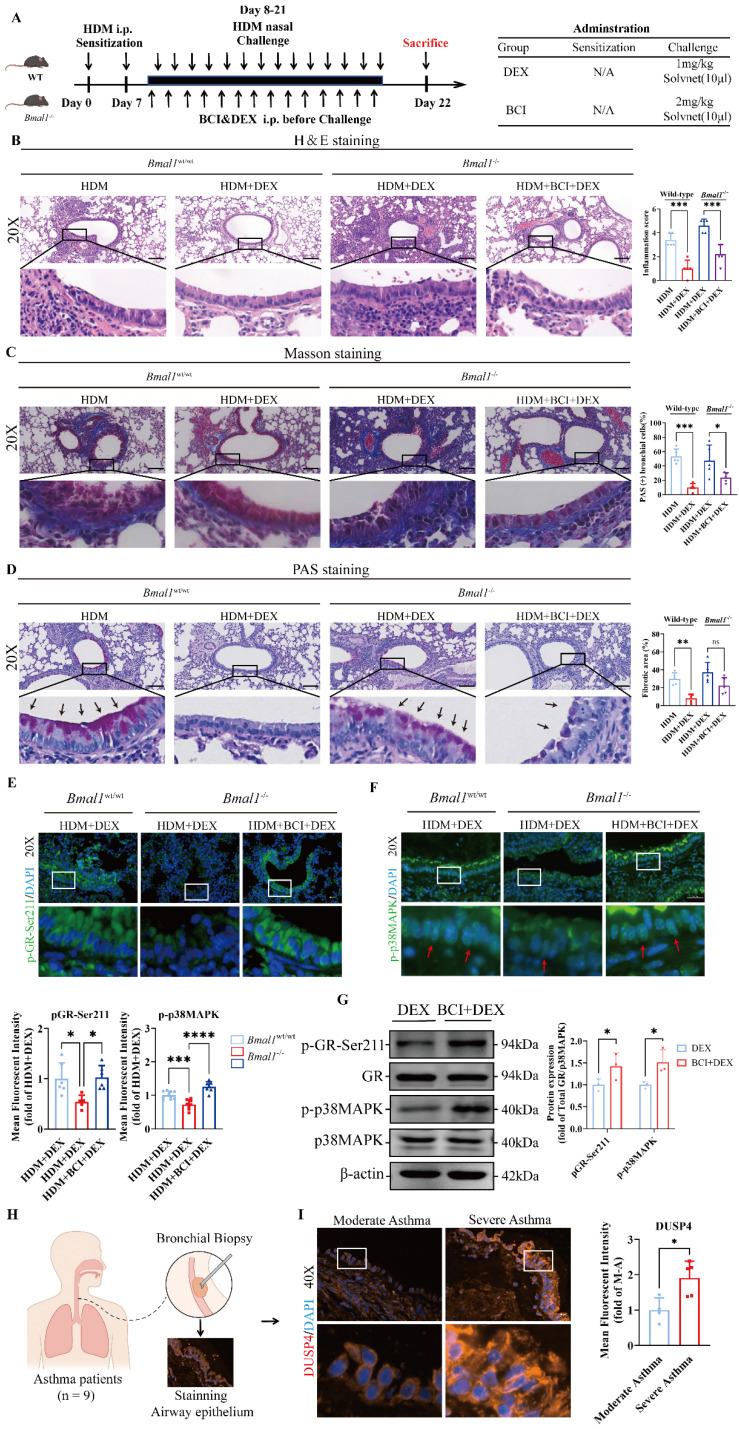
** Inhibition of DUSP4 restored GC sensitivity in BMAL1-deficient airway epithelial cells. (A)** Schematic diagram of the experimental protocol for HDM sensitization, followed by HDM challenge and treatment with DEX or BCI + DEX. **(B)** Representative images of H&E-stained lung tissue sections from Bmal1^-/-^ mice in different treatment groups. (Scale bar = 100 μm). **(C-D)** The amount of collagen around the airways and percentages of PAS-positive airway epithelial cells were quantified (scale bar = 100 μm). **(E-F)** Immunofluorescence staining demonstrated the phosphorylation levels of GR-Ser211 and p38MAPK in airway epithelial cells (scale bar = 50 μm). **(G)** Western blot analysis of p-GR-Ser211 and p-p38MAPK in BMAL1-deficient HBECs treated with DEX or BCI + DEX. **(H)** Schematic diagram of bronchial biopsy and immunofluorescence staining from an asthma patient. **(I)** Immunofluorescence staining demonstrated the levels of DUSP4 in airway epithelium (Scale bar = 50 μm). *P<0.05, **P<0.01, ***P<0.001, ****P<0.0001.

To further validate the therapeutic potential of DUSP4 inhibition *in vivo*, we administered the DUSP4 inhibitor BCI to *Bmal1*^-/-^ mice with HDM-induced asthma in combination with DEX treatment (Fig 8A). Histological analysis revealed that airway inflammation, mucus production, and remodeling were significantly alleviated in the BCI + DEX treatment group compared to DEX monotherapy in *Bmal1*^-/-^ mice (Fig 8B-D). Immunofluorescence staining further revealed that the levels of p-GR-Ser211 and p-p38MAPK in airway epithelial cells were substantially restored in the BCI + DEX group (Fig 8E-F), indicating functional reactivation of the p38MAPK-GR pathway *in vivo*.

Western blot analysis consistently revealed that, in BMAL1-deficient HBECs, BCI significantly enhanced p38MAPK phosphorylation and GR-Ser211 phosphorylation relative to DEX monotherapy. (Fig 8G). Similarly, in the SD mouse model, BCI restored the efficacy of DEX and enhanced the phosphorylation of p38MAPK and GR-Ser211 (Fig S6). These data demonstrate that DUSP4 inhibition can effectively reverse the impaired GR phosphorylation and p38MAPK activity induced by BMAL1 loss. Furthermore, immunofluorescence staining showed higher DUSP4 expression in airway epithelium from patients with severe asthma compared with those with moderate asthma (Fig 8H-I), suggesting DUSP4 may contribute to the pathogenesis and severity of asthma.

Collectively, our findings suggest that pharmacological targeting of DUSP4 restores GC responsiveness by reactivating the p38MAPK-GR axis, offering a potential therapeutic strategy for managing GC resistance in the context of circadian disruption.

## Discussion

Despite their widespread use, GCs are ineffective in some asthma patients, creating a considerable clinical problem 3. While airway inflammation and GC resistance have been linked to multiple mechanisms, increasing evidence implicates circadian rhythm disruption as a contributing factor 24, yet the molecular link between circadian clock perturbation and GC signaling remains unclear. In this study, using BMAL1-knockdown HBECs and a well-established *Bmal1* knockout mice model 46, 47, we identify the circadian transcription factor BMAL1 as a critical regulator of GC responsiveness in airway epithelial cells, acting through a DUSP4-p38MAPK-GR axis. Our findings showed that BMAL1 dysfunction exacerbates HDM-induced airway inflammation and promotes GC resistance by impairing GR-Ser211 phosphorylation and downstream anti-inflammatory responses.

Our clinical and animal data support a direct association between circadian misalignment and asthma severity. Asthmatic patients with a late phenotype exhibited poorer disease control and higher exacerbation rates, correlating with increased daily GC consumption. In parallel, mice subjected to sleep deprivation, a model of circadian disruption, showed exacerbated HDM-induced airway inflammation, accompanied by downregulation of BMAL1 in airway tissues. These findings are consistent with previous studies linking altered circadian rhythms to asthma exacerbations 48, 49, and underscore the functional relevance of peripheral clocks within the airway epithelium 50. Notably, BMAL1 was predominantly expressed in airway epithelial cells and rhythmically regulated under physiological conditions, but this oscillation was attenuated following HDM exposure. These findings indicate that BMAL1 acts as a suppressor of inflammation associated with asthma, consistent with previous reports 51, 52.

Mechanistically, we showed that BMAL1 deficiency in airway epithelial cells enhanced activation of NF-κB and AP-1, key pro-inflammatory transcription factors whose inhibition represents a major anti-inflammatory action of GCs 35. Importantly, we also observed that BMAL1 deficiency was associated with elevated T1 inflammatory cytokines in knockout mice, suggesting a potential role of BMAL1 loss in driving asthma endotype switching. A growing number of studies link GC insensitivity to disturbed GR function 38, 53, 54. In BMAL1-deficient HBECs and mice, GC-mediated suppression of inflammatory cytokines was attenuated, and expression of GC-responsive genes such as GILZ and FKBP5 was diminished, suggesting impaired GR signaling.

Normal cellular processes and disease development are influenced by posttranslational modifications (PTMs), which are one of the most common types of modification affecting protein function. PTMs of the GR protein are primarily regulated by phosphorylation, as stated in previous studies 19, 21. Previous studies have indicated that insufficient phosphorylation of GR significantly impairs the anti-inflammatory effect of GC, suggesting that GR phosphorylation is a major cause of GC resistance 20. Importantly, *BMAL1* deletion led to decreased phosphorylation of GR at Ser211, which is a critical post-translational modification required for GR activation 40. These findings suggest that circadian disruption not only promotes inflammation but also directly interferes with GC-GR signaling.

Although MAPK pathways have been implicated in GR regulation, their role in GC resistance is complex. Excessive MAPK activity can ultimately lead to impaired GR function in severe asthma and COPD 5, 55, 56. However, basal p38MAPK activity is required for GR-Ser211 phosphorylation and GC function 57, 58. A study demonstrated that the activation of the MAPK pathway resulting in increased p-GR-Ser211 levels, can significantly improve the GC sensitivity in leukemic lymphoid CEM cells, which are known for their poor responsiveness to GC 59. Another study in asthma revealed that enhancing the phosphorylation of Ser211 can restore the responsiveness of airway smooth muscle cells to GCs 60. We found that BMAL1 knockdown selectively suppressed p38MAPK activation without suppressing ERK or JNK pathways. These effects were associated with reduced GR phosphorylation and GC responsiveness, reinforcing the notion that the p38MAPK pathway plays a dual role in balancing inflammatory and GC signaling. Interestingly, we also observed that the ERK pathway, another pathway associated with asthma, was significantly activated after BMAL1 knockdown. The significance of the ERK pathway in circadian disruption-related asthma is under investigation and will be addressed in our future studies. Nevertheless, the diverse expression patterns of MAPKs and the intricate interplay and regulatory loops present challenges for pharmacological interventions.

We further identified DUSP4, a MAPK phosphatase 44, as a direct transcriptional target of BMAL1. BMAL1 overexpression suppressed DUSP4 expression, while ChIP and luciferase reporter assays confirmed BMAL1 binding to the DUSP4 promoter. Functional studies showed that disrupting the DUSP4 promoter restored p38MAPK phosphorylation in BMAL1-deficient cells, supporting a mechanistic model in which BMAL1 maintains basal p38MAPK activity by repressing DUSP4 transcription.

To validate the therapeutic relevance of this mechanism, we used the DUSP4 inhibitor BCI in *Bmal1*-deficient and SD model mice. Remarkably, BCI combined with DEX restored GR-Ser211 phosphorylation, suppressed airway inflammation, and reversed GC resistance both *in vivo* and *in vitro*. Consistently, human bronchial biopsy specimens revealed elevated DUSP4 expression in airway epithelial cells from patients with severe asthma compared with those with moderate asthma, further supporting the clinical relevance of this pathway.

These findings suggest the DUSP4-p38MAPK-GR axis as a potential therapeutic target for restoring GC sensitivity in asthma with circadian disruption. While BCI is currently a tool compound with limited specificity and pharmacokinetic properties, our results lay the groundwork for developing DUSP4-targeted therapies in SRA.

### Limitations

Although targeting the DUSP4-p38MAPK-GR could be a potential therapy to restore GC sensitivity, certain limitations of this study should be considered. For *in vitro* assays, immortalized monolayer HBEC cultures were utilized instead of primary pseudostratified columnar epithelium, which more closely models airway physiology, inclusive of ciliary activity and mucus production Field 61. Additionally, our primary focus was on dysregulated GC signaling in airway epithelial cells, and our prior work demonstrated that other immune cells, specifically innate lymphoid cell type 3 (ILC3) cells, T helper type 17 (Th17) cells, and M1-polarized macrophages, also play a role in GC resistance during circadian disruption 61. Future research will emphasize a more detailed analysis of the immune microenvironment, specifically the function of immune cell subtypes in GC resistance. Despite the widespread use of the SD-mouse model in circadian disruption studies, it mostly reflects behavioral rhythm disturbances and offers an incomplete representation of central circadian clock dysfunction. Future studies incorporating SCN-targeted analyses or clock gene reporter systems may help clarify the direct role of central clocks in asthma pathogenesis.

## Conclusion

In conclusion, our findings provide evidence for a critical role of the circadian clock in GC resistance in asthma. Normalizing circadian oscillations is an important approach for restoring GC sensitivity. In addition, combination therapies comprising clock-normalizing agents and other modalities, such as conventional GC therapy or biologic therapy, may have either additive or synergistic effects. Based on our results, we suggest that restoring circadian clock function may offer a novel therapeutic strategy for asthma management.

## Methods

### Clinical data collection from patients with asthma

Clinical data were collected from patients with asthma who were admitted to the Department of Respiratory and Critical Care Medicine, Nanfang Hospital, Southern Medical University from September 2023 to September 2024. Diagnosis, grading and treatment of asthma patients were based on GINA2023. The demographic and functional characteristics of all the study subjects are shown in Table S1. All participants provided informed consent and independently completed the Munich Time Type Questionnaire as described by Roenneberg, T.'s study 62. The MEQ-SA consists of 19 items evaluating an individual's preferred timing for sleep and activity, and participants were classified into as early chronotype (scores≥59) and late chronotype (<41). The ACT includes 5 items related to asthma symptoms, activity limitation, and medication use over the past 4 weeks. Total scores range from 5 to 25. According to GINA2023, patients with ACT scores ≤19 were considered to have uncontrolled asthma, while scores >19 indicated controlled asthma.

### Materials and reagents

House dust mites (HDM) were purchased from ALK-Abelló A/S. Dexamethasone acetate (S3124) was obtained from Selleck Chemicals (USA). Anti-BMAL1 (14268-1-AP), anti-cJUN (24909-1-AP), and anti-FOS (66590-1-IG) antibodies were obtained from Proteintech Technology (China). Anti-phospho-GR-Ser211 (4161), anti-GR (3660), anti-phospho-p38MAPK (4511), anti-p38MAPK (8690), and DUSP4 (5149) and DUSP6 (50945) were purchased from Cell Signaling Technology (CST, USA). Anti-phospho-NFκB p65 (sc-136548) and anti-NFκB p65 (sc-8008) antibodies were obtained from Santa Cruz Biotechnology (USA). Anti-β-actin (66009-1-IG) and anti-α-tubulin (11224-1-AP) were used as loading controls from Proteintech Technology.

### Cell culture and transfection

The human bronchial epithelial cell line (HBE-135, ATCC) was cultured as previously described 63, 64. HBECs were treated with DEX (10μM) for 12 h prior to stimulation with HDM (400U/mL) for 24 h. For BCI treatment, cells were pretreated with BCI (2μM, MCE, USA) for 24 h before DEX stimulation. Transfection of the siRNAs or plasmids was performed using Lipo3000 and P3000 (Thermo Fisher Scientific). HBECs were transiently transfected with siRNA or scrambled siRNA negative control (NC) designed by HanYi Biosciences Inc (China). After 12 h, the medium was changed for the following experiments. The siRNA target sequences used are shown in Table S2**.** The OE-BMAL1 and DUSP4 mutant (Mut1 and Mut2) plasmids were designed and purchased from Genechem (China).

### Quantitative real‑time polymerase chain reaction (qRT‑PCR)

Total RNA was extracted using TRIzol reagent following the manufacturer's protocol. Complementary DNA (cDNA) was synthesized by reverse transcription using the Accurately Biology kit (AG11706). Quantitative PCR was conducted with SYBR Master Mix (GDSBio, P2105) on a Bio-Rad real-time PCR system. Primer sequences for target gene amplification are listed in Table S3. Relative mRNA levels were determined using the 2^-∆∆Ct^ method and normalized to GAPDH.

### Western blot

Equal amounts of protein from HBECs were used for Western blot. Proteins were separated on SDS-PAGE gels and transferred to nitrocellulose membranes. After blocking in 5% bovine serum albumin for 2 h, they were incubated with primary antibodies followed by fluorescently labeled secondary antibodies (Invitrogen) and then imaged using the Li-COR Odyssey system.

### Asthma animal model and treatments

*Bmal1*^+/-^ mice were provided by View Solid Biotech (China). *Bmal1*^-/-^ and *Bmal1*^+/+^ mice were confirmed by genotyping of ear tissue. Genotyping was performed via PCR using primers OL2646: 5′-CCACCAAFCCCAFCAACRCA-3′; OL2657: 5′-ATTCGGCCCCCTATCTTCTGC-3′; and OL278: 5′-TCGCCTTCTATCGCCTTCTTCTTGACG-3′. The results of the PCR identification are shown in the Supplementary Figure (Fig S7A-B). C57BL/6 wild-type mice were purchased from the Laboratory Animal Center, Southern Medical University. The mice were maintained on a 12 h light/dark cycle with free access to food and water. For the experimental asthma model, mice were injected intraperitoneally with 100μL allergen mixture containing HDM (4000U/mice) on day 0 and day 7, and were challenged by intranasal administration of the 20μL allergen mixture (400U/mice) daily for 14 days as described previously 64. The mice received DEX (1 mg/kg) intraperitoneally 1 h before each HDM challenge. In the BCI treatment group, mice were additionally administered BCI (2 mg/kg) via intraperitoneal injection prior to DEX administration on each challenge day. All the mice were sacrificed on day 22 for further analysis. All experiments were performed in accordance with protocols approved by the Institutional Animal Care and Use Committee of Southern Medical University.

### Chronic sleep deprivation

The custom-made device (XR-XS108, shxinruan, China) was used to induce sleep disruption in rodents. During the sweeping movement, mice were forced to step over a moving sweeper to remain awake. SD was induced during a 16-hour cycle from 8:00 am (ZT0) to 12:00 am (ZT16) for 7 or 14 days, and mice had *ad libitum* access to food and water.

### Immunofluorescence microscopy

HBECs were gently washed with PBS, fixed in 4% paraformaldehyde, permeabilized with 0.3% Triton-X-100 for 20 min, blocked with 3% BSA for 1 h at room temperature, and incubated overnight with primary antibodies at 4°C. Immunodetection was performed with the following primary antibodies: anti-phospho-NF-κB p65-Ser536 (1:1000; 3033S, CST), anti-phospho-GR-Ser211 (1:1500; 4161, CST), anti-phospho-p38MAPK (1:500; 4511, CST), anti-cJUN (1:100, 24909-1-AP, Proteintech). The samples were subsequently incubated with Alexa Fluor 594 anti-Rat (1:100; A-21209, Life Technologies), Alexa Fluor 488 anti-mouse (1:100; A-11001, Life Technologies) and Alexa Fluor 488 anti-Rabbit (1:100; A31628, Life Technologies) antibodies at room temperature for 2 h. The nuclei were stained with DAPI (Beyotime) for 5 min. Fluorescence signals were captured with identical settings across groups, with an exposure time of 500 ms.

### Analysis of bronchoalveolar lavage fluid and serum

Bronchoalveolar lavage fluid (BALF) samples were collected by lavaging the right lung with 1.4 mL PBS. After estimating the total number of cells using a hemocytometer (Bio-Rad), BALF was centrifuged at 1200 rpm for 10 min, and the cell pellet was stained with Modified Giemsa (Beyotime) and a total of 200 cells were counted and classified. The concentrations of cytokines (IL-4, IL-5, IL-13, TSLP, IL-25, IL-33, IFN-γ, IL-6) in the BALF supernatant were measured using ELISA kits (Cusabio). Blood samples were collected, centrifuged at 3000 g for 10 min and assayed for HDM-specific IgE using an ELISA kit (Cusabio).

### Histotechnology

Paraffin-embedded lung tissues were cut into 5-µm-thick sections, deparaffinized with xylene and rehydrated with sequential steps in graded ethanol series (98-95-70%). Lung sections were stained with H&E (Beyotime), Masson (Solarbio), PAS (Solarbio), and phospho-GR-Ser211 antibody (1:200; 4161, CST) respectively and were examined with a digital camera. Inflammatory infiltration was scored as follows: 0 for absence of inflammatory cells; 1 for sparse infiltration; 2 when inflammatory cells formed a ring one cell layer thick; 3 for rings 2-4 cells thick; and 4 for rings exceeding 4 cell layers in thickness 65.

### Chromatin immunoprecipitation

Chromatin immunoprecipitation (ChIP) experiments were performed using the BeyoChIP™ Enzymatic ChIP Assay Kit (P2083S, Beyotime) according to the manufacturer's recommendations. Chromatin was immunoprecipitated with immunoglobulin G (2729, CST) or anti-BMAL1 (1:50; 14020, CST). 2% of the total DNA served as input control. DNA enrichment in ChIP samples was quantified by qPCR, with primer sequences detailed in Table S4.

### Dual-luciferase assay

The luciferase pGL3 plasmids were transfected into HBECs and incubated for 48 h. Reporter activity was quantified using the Dual-Luciferase® Reporter Assay System (Promega, E2909, China) and normalized to Renilla luciferase signals.

### RNA sequencing and analysis

Total RNA was extracted using TRIzol reagent as previously described. Polyadenylated RNA was enriched from 1 μg total RNA with Dynabeads Oligo (dT) 25-61005 (Thermo Fisher) through two purification steps. The purified poly(A) RNA was fragmented at 94°C for 5-7 min using a Magnesium RNA Fragmentation Module (NEB). First-strand cDNA was synthesized with SuperScript™ II Reverse Transcriptase (Invitrogen, 1896649), followed by second-strand synthesis incorporating dUTP using E. coli DNA polymerase I (NEB, m0209), RNase H (NEB, m0297), and dUTP solution (Thermo Fisher, R0133). An A-base is then added to the blunt ends of each strand to prepare them for ligation to the indexed adapters. Each adapter contains a T-base overhang for ligation of the adapter to the A-tailed fragmented DNA. Single or dual index adapters are ligated to the fragments and size selection was performed using AMPureXP beads. After heat-labile UDG enzyme (m0280, NEB) treatment of the U-labeled second-stranded DNAs, the ligated products are amplified by PCR under the following conditions: initial denaturation at 95°C for 3 min; 8 cycles of denaturation at 98°C for 15 s, annealing at 60°C for 15 s, and extension at 72°C for 30 s; and then final extension at 72°C for 5 min. The average insert size of the final cDNA library was 300 ± 50 bp. Finally, 2×150 bp paired-end sequencing (PE150) was performed on an Illumina Novaseq™ 6000 (LC-Bio Technology CO., Ltd) following the manufacturer's recommended protocol.

### GEO analysis

Datasets (GSE74986) were downloaded from the GEO database (https://www.ncbi.nlm.nih.gov/geo/) in MINiML format. Box plots were generated using the “ggplot2” and “pheatmap” packages implemented in R software (version 4.3.0).

### Phosphorylation site and kinase prediction

Experimentally validated phosphorylation sites of GR were identified using PhosphoSitePlus®, a curated database of post-translational modifications based on published mass spectrometry and biochemical data. Potential upstream kinases targeting GR phosphorylation were predicted with GPS 6.0 using default parameters, focusing on Ser211 to aid the identification of signaling pathways potentially regulated by BMAL1.

### Bronchial biopsy specimens

Bronchial biopsy samples were collected from patients with asthma at the First Affiliated Hospital of Guangzhou Medical University between January 2024 and June 2025. Asthma severity was classified according to the GINA2023 guidelines, and all diagnoses and grading were confirmed by associate chief physicians or above.

### Statistical analysis

Prism 9.0 software (GraphPad) was used for data analysis. All experiments were conducted with at least three independent replicates. Student's t-test was used to analyze differences between 2 groups, and one-way ANOVA was used to compare differences between more than 2 groups. P values less than 0.05 were considered statistically significant.

## Supplementary Material

Supplementary figures and tables.

## Figures and Tables

**Table 1:** Baseline information on patients with asthma

	Total (n=70)	Early chronotype (n=34)	Late chronotype (n=36)	P-value
**MEQ-SA score**		≥59	<41	
**Age**				0.755
Mean (SD)	43.557 (13.671)	42.941 (14.973)	44.139 (12.504)	
**Gender, n (%)**				0.863
Female	46 (65.7)	22 (64.7)	24 (66.7)	
Male	24 (34.3)	12 (35.3)	12 (33.3)	
**Asthma control test (ACT) , n (%)**				0.024*
Controlled (20-25)	32 (45.7)	21 (61.8)	11 (30.6)	
Partly controlled (16-19)	17 (24.3)	7 (20.6)	10 (27.8)	
Uncontrolled (5-15)	21 (30.0)	6 (17.6)	15 (41.7)	
**Annual exacerbations, n (%)**				0.043*
0	36 (51.4)	22 (64.7)	14 (38.9)	
1	22 (31.4)	10 (29.4)	12 (33.3)	
2	8 (11.4)	2 (5.88)	6 (16.7)	
≥3	4 (5.71)	0 (0)	4 (11.1)	
**Annual medical appointments, n (%)**				0.117
Mean (SD)	7.871 (6.251)	6.735 (5.534)	8.944 (6.761)	
**Daily hormonal inhalation, n (%) (Beclometasone)**				0.581
200μg	12 (17.1)	5 (14.7)	7 (19.4)	
320μg	43 (61.4)	23 (67.6)	20 (55.6)	
1000μg	15 (21.4)	6 (17.6)	9 (25.0)	
Mean (SD)	445.123 (295.192)	422.353 (274.702)	466.667 (315.685)	0.8497

Wilcoxon rank sum test; Pearson's Chi-squared test; Fisher's exact test. *P<0.05

## Abbreviations

BMAL1: Brain and muscle ARNT-like 1
GC: Glucocorticoid
GR: Glucocorticoid receptor
SD: Sleep deprivation
NF-κB: Nuclear factor kappa-B
AP-1: Activating protein-1
HDM: House dust mite
MAPK: Mitogen-activated protein kinase
ERK: Extracellular regulated protein kinases
JNK: cJUN N-terminal kinase
MPAS: MAPK Pathway Activity Score
DUSP: Dephosphatase dual specificity protein phosphatase
SRA: Steroid resistant asthma
IL: Interleukin
IFN: Type I interferon
DEX: Dexamethasone
HBEC: Human bronchial epithelial cell
BALF: Bronchoalveolar lavage fluid
EOS: Eosinophil
NEU: Neutrophil
LYM: Lymphocyte
TSLP: Thymic stromal lymphopoietin
