# Supplementary material for: Disruption of the Clock Component BMAL1 in HDM-induced Asthma Causes GC Resistance in Airway Epithelial Cells by Regulating GR Phosphorylation through the DUSP4-p38MAPK Pathway
Source: Int J Biol Sci. 2025 Oct 10;21(14):6482–500. doi: 10.7150/ijbs.119486 (PMC12594601; doi:10.7150/ijbs.119486)
Supplement: Supplementary file 1 — Supplementary figures and tables. [file ijbsv21p6482s1.pdf]

Supplemental Table 1: The detailed clinical case information of 70 patients with asthma are listed.

Pathological ID	Age	Gender	Sleeping type	Asthma control test (ACT)	Annual exacerbation	Annual medical appointment	Daily hormonal inhalation (Beclometasone, ug)
0006864280	24	Male	Early chronotype	14	1	1	320
0000144702	40	Female	Early chronotype	15	2	5	200
0002823147	69	Male	Early chronotype	14	2	5	1000
0000290983	57	Female	Early chronotype	13	1	7	320
0005784391	33	Male	Early chronotype	15	1	2	320
0000156039	54	Female	Early chronotype	14	1	7	320
0000286863	54	Female	Early chronotype	18	1	10	320
0000895744	26	Female	Early chronotype	17	1	3	200
0000565416	37	Male	Early chronotype	19	1	1	320
0005784391	33	Male	Early chronotype	17	1	2	320
0000186507	39	Female	Early chronotype	19	1	15	320
0005316041	45	Male	Early chronotype	19	1	17	320
0000630960	68	Female	Early chronotype	18	0	1	320
0001057733	25	Female	Early chronotype	25	0	2	320
0000901813	50	Male	Early chronotype	24	0	18	320
0000915964	67	Female	Early chronotype	25	0	5	320
0000431656	36	Female	Early chronotype	23	0	11	200
0000299899	27	Female	Early chronotype	24	0	2	1000
0006761152	21	Male	Early chronotype	25	0	3	320
0006585485	65	Female	Early chronotype	25	0	6	200
0007017124	18	Male	Early chronotype	25	0	1	320
0005505311	21	Female	Early chronotype	25	0	7	200
0000170978	45	Female	Early chronotype	24	0	5	320
0005174004	42	Male	Early chronotype	23	0	7	320
0000032868	50	Female	Early chronotype	25	0	2	320
0000488946	50	Female	Early chronotype	25	0	5	1000
0000067951	45	Female	Early chronotype	23	0	10	1000
0003864735	49	Female	Early chronotype	25	0	9	320
0000389597	51	Female	Early chronotype	25	0	25	320
0000123669	38	Female	Early chronotype	25	0	7	320
0000797264	49	Male	Early chronotype	25	0	10	1000
0000353760	65	Male	Early chronotype	24	0	10	1000
0006854399	49	Female	Early chronotype	24	0	2	320
0006784418	18	Female	Early chronotype	24	0	6	320
0000596274	55	Female	Late chronotype	14	3	24	1000
0000253082	47	Female	Late chronotype	14	3	12	200
0000204668	59	Female	Late chronotype	15	3	26	1000
0005143077	32	Female	Late chronotype	14	3	4	200

0000424727	46	Female	Late chronotype	13	1	5	320
0000371152	28	Female	Late chronotype	15	2	3	320
0006783498	60	Male	Late chronotype	14	2	3	200
0005296926	20	Female	Late chronotype	12	1	3	320
0002722309	26	Male	Late chronotype	14	1	4	320
0000100065	51	Male	Late chronotype	15	1	7	1000
0003820105	32	Female	Late chronotype	15	2	3	320
0001002811	26	Female	Late chronotype	13	1	7	320
0006312271	30	Female	Late chronotype	14	1	3	320
0000894959	48	Male	Late chronotype	13	2	3	200
0000974874	63	Female	Late chronotype	13	2	29	200
0000837754	59	Male	Late chronotype	16	1	9	320
0005037468	29	Female	Late chronotype	16	1	6	320
0000221555	42	Female	Late chronotype	18	1	7	320
0000778947	49	Male	Late chronotype	19	1	4	320
0002158738	44	Female	Late chronotype	16	2	14	1000
0005316041	45	Male	Late chronotype	16	1	17	320
0002158738	44	Female	Late chronotype	18	1	10	320
0000406170	42	Female	Late chronotype	19	0	15	1000
0002286897	43	Male	Late chronotype	19	0	5	320
0000555819	55	Female	Late chronotype	19	0	10	320
0000383916	45	Female	Late chronotype	25	0	9	320
0000176053	49	Female	Late chronotype	25	0	5	320
0005702177	18	Female	Late chronotype	24	0	4	320
0000195810	35	Female	Late chronotype	24	0	17	320
0000286956	39	Male	Late chronotype	25	0	6	320
0000504664	45	Female	Late chronotype	24	0	8	200
0000433860	62	Male	Late chronotype	24	0	14	1000
0000210073	54	Female	Late chronotype	25	0	11	1000
0001661128	66	Male	Late chronotype	24	0	2	200
0002016368	56	Male	Late chronotype	25	0	7	1000
0002032135	45	Female	Late chronotype	25	0	6	1000

Supplemental Table 2. The siRNA sequences of the primers used are listed.

Gene Symbol	Primer Sequence
BMAL1-siRNA1	F:UCUAGGCACAUCGUGUUAUTT<br>R:AUAACACGAUGUGCCUAGATT
BMAL1-siRNA2	F:ACGCGAUAGAUGGAAAGUUTT<br>R:AACUUUCCAUCUAUCGCGUTT
BMAL1-siRNA3	F:AGAAUGUCAUAGGCAAGUUTT<br>R:AACUUGCCUAUGACAUUCUTT

Supplemental Table 3. The sequences of the primers used for qPCR are listed.

Gene Symbol	Primer Sequence
BMAL1	F:CCAAGAAAGTATGGACACAGACAAA<br>R:GCATTCTTGATCCTTCCTTGGT
IL-25	F:CAGGTGGTTGCATTCTTGGC<br>R:GAGCCGGTTCAAGTCTCTGT
IL-33	F:GTGACGGTGTTGATGGTAAGAT<br>R:AGCTCCACAGAGTGTTCCTTG
TSLP	F:ATGTTCGCCATGAAAACTAAGGC<br>R:GCGACGCCACAATCCTTGTA
GILZ	F:GGACTTCACGTTTCAGTGGACA<br>R:AATGCGGCCACGGATG
FKBP5	F:CCATTGCTTTATTGGCCTCT<br>R:GGATATACGCCAACATGTTCAA
GR-α	F:TGACTCTACCCTGCATGTACGACCA<br>R:TCAGCTAACATCTCGGGGAATTCAA
GR-β	F:GAAGGAAACTCCAGCCAGAA<br>R:CCACATAACATTTTCATGCATAGA
DUSP4	F:ACCCAGAAGACTGTGGATGG<br>R:GTCGGCCTTGTGGTTATCTTC
DUSP5	F:GCCAGCTTATGACCAGGGTG<br>R:GTCCGTCGGGAGACATTCAG
DUSP6	F:GAAATGGCGATCAGCAAGACG<br>R:CGACGACTCGTATAGCTCCTG
DUSP12	F:CTGGGGTCGAGGATCTATGG<br>R:CGGTCCAGATGGCTGAGTAG
DUSP16	F:AGGTGGGTTTGCTGAGTTCTC<br>R:TTCTAGACGGCAGGTCAGGT
β-actin	F:CATGTACGTTGCTATCCAGGC<br>R:CTCCTTAATGTCACGCACGAT

M-Bmal1	F:ACAGTCAGATTGAAAAGAGGCG<br>R:GCCATCCTTAGCACGGTGAG
M-β-actin	F:GTGACGTTGACATCCGTAAAGA<br>R:GCCGGACTCATCGTACTCC

Supplemental Table 4. The sequences of the primers used for CHIP are listed.

Gene Symbol	Primer Sequence
DUSP4-1436-1445	F:GCGCCAAAGGAAATAGCCG<br>R:AACCGGGAAAAACCTACGGG
DUSP4-1768-1777	F:GAGTTTTCTCCTCGGCTTAGAGG<br>R:AAAACCTACGGGGCTGTCAC

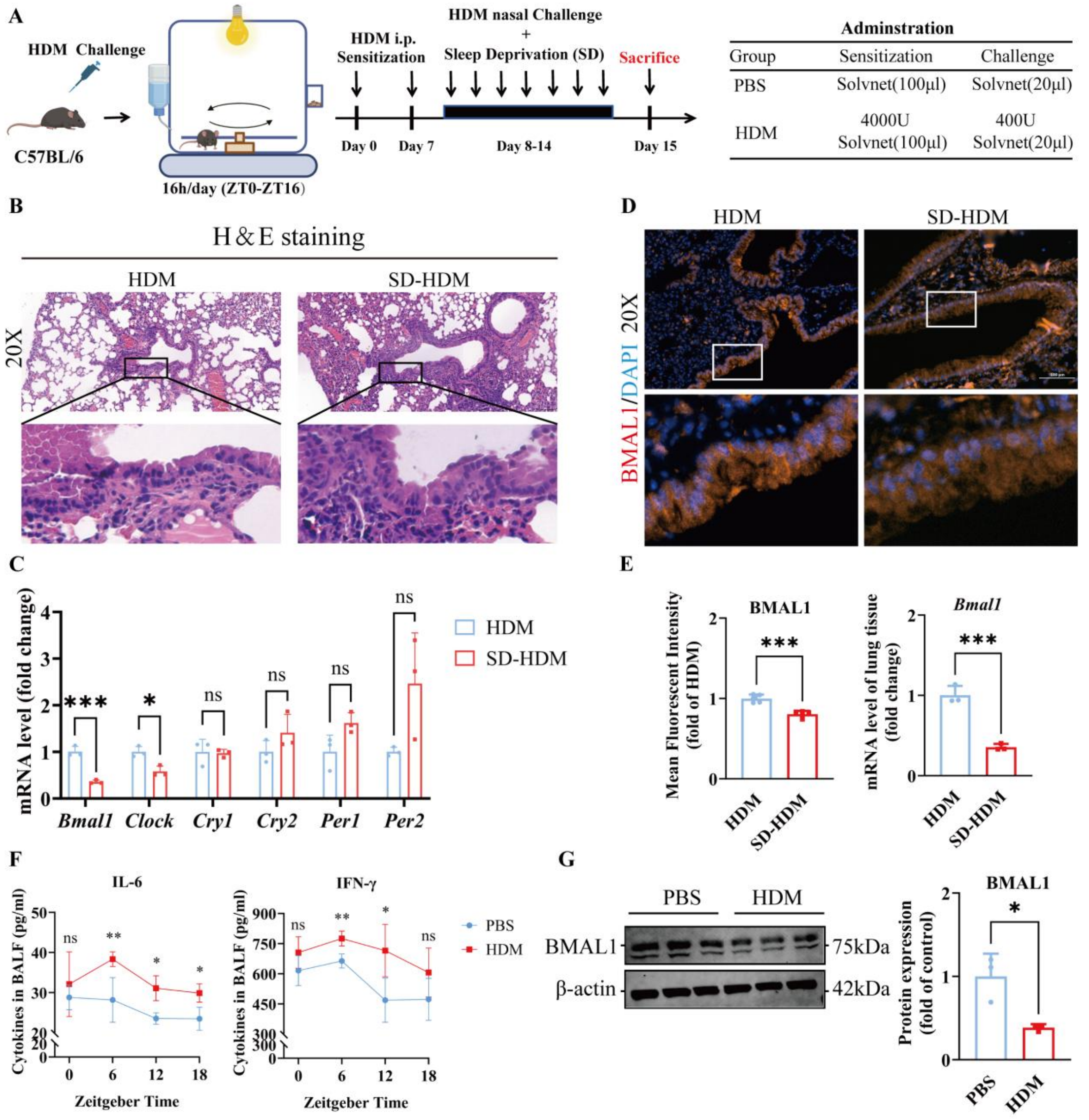

Supplemental Figure 1. Sleep deprivation impairs circadian clock gene expression and increases airway inflammation in HDM-induced asthma mice

(A) Schematic diagram of the SD mouse model. **(B)** Representative images of H&E-stained lung tissue sections from the different groups (scale bar =100 μm). **(C)** The mRNA level of *Bmal1*, *Clock*, *Cry1*, *Cry2*, *Per1* and *Per2* in mouse lung tissues. **(D)** Protein expression of BMAL1 in mouse lung tissues, as determined by immunofluorescence staining (scale bar =100 μm) **(E)** The expressionl of BMAL1 in mouse lung tissues. **(F)** The levels of IL-6 and IFN-γ in BALF by ELISA. **(G)** The

protein expression of BMAL1 in HBECs by western blotting.

**P*<0.05, ***P*<0.01, ****P*<0.001, *****P*<0.0001

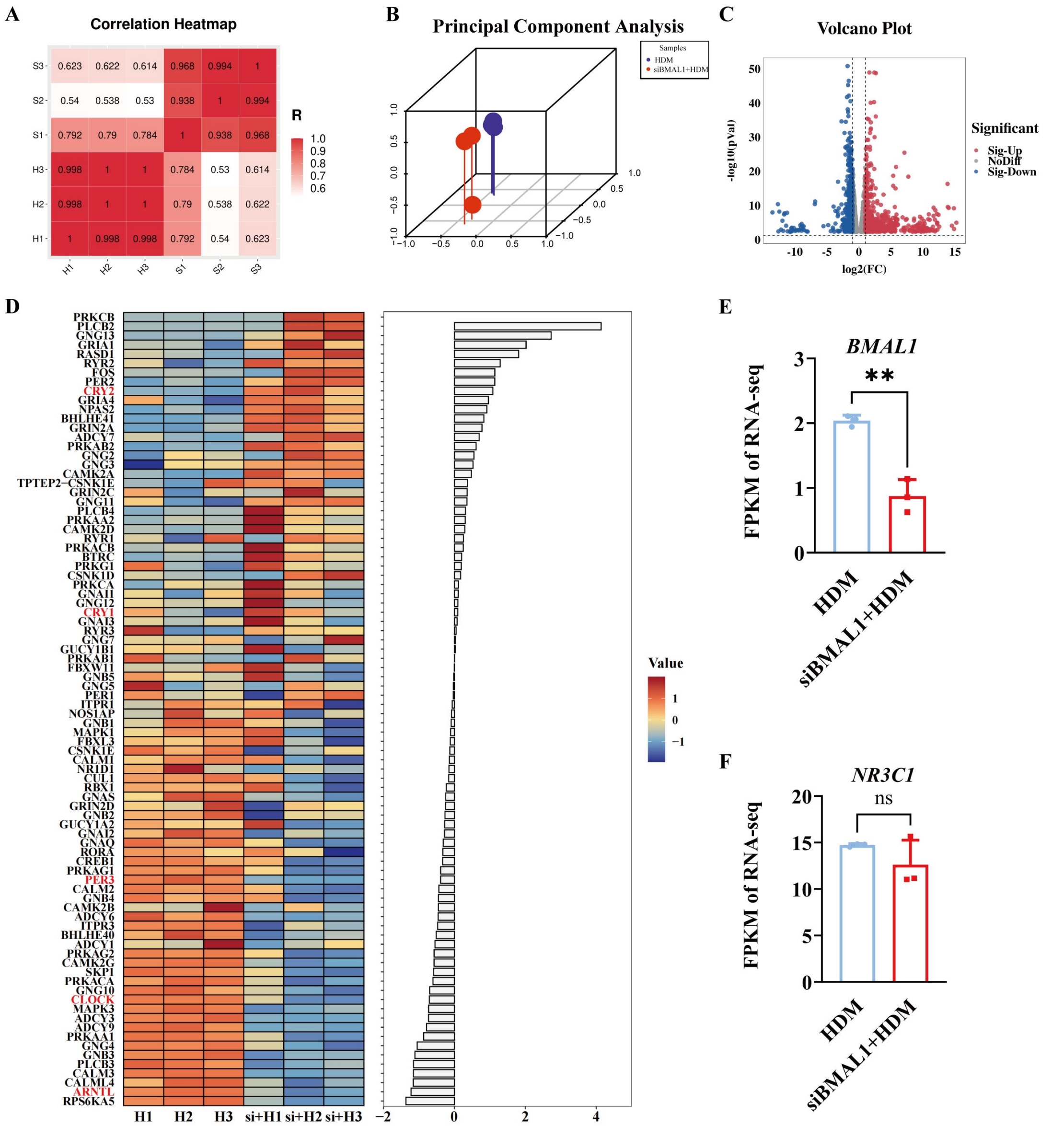

Supplemental Figure 2. The expression levels of circadian clock gene in HBECs after knockdown of BMAL1.

(A) Pearson's correlation analysis between two groups. **(B)** Weighted uniFrac-based PCA of RNA expression between two groups. **(C)** Volcano plots showing differences in gene expression. Red indicates significantly upregulated genes, and blue indicates significantly downregulated genes. **(D)** Heatmap demonstrated the transcription level of targeting circadian rhythm-related components. **(E-F)** The expression of *BMAL1*

and *NR3C1* in HBECs from RNA-seq.

**P<0.01

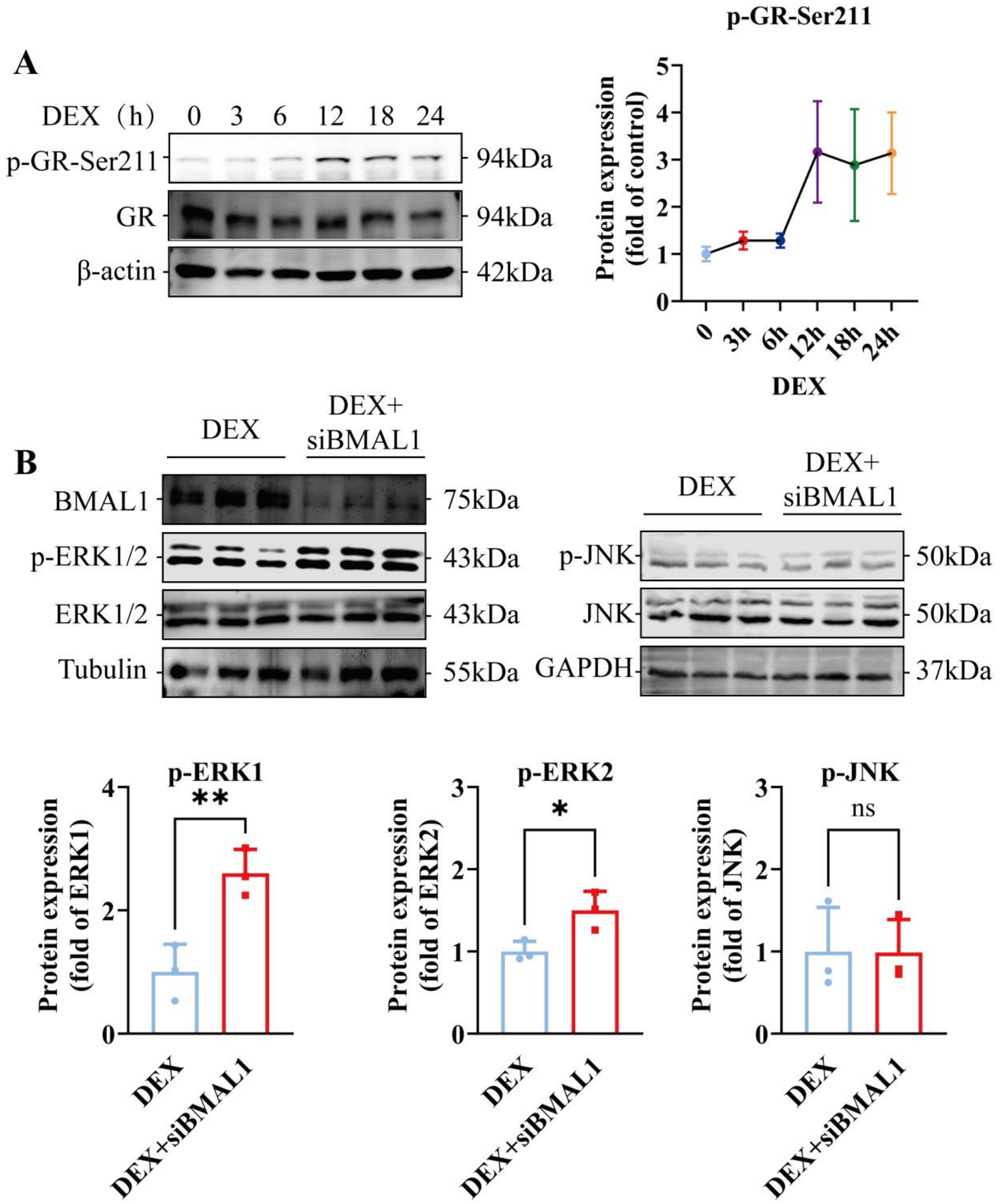

Supplemental Figure 3. Phosphorylation of the GR-Ser211 after DEX treatment and activation of the MAPK pathway after BMAL1 knockdown.

(A) The protein expression of p-GR-Ser211 in HBECs with gradient concentrations DEX treatment. **(B)** Activation of the ERK pathway in HBECs after knockdown of BMAL1 by western blotting. **(C)** Activation of the JNK pathway in HBECs.

**P*<0.05, ***P*<0.01, ****P*<0.001, *****P*<0.0001

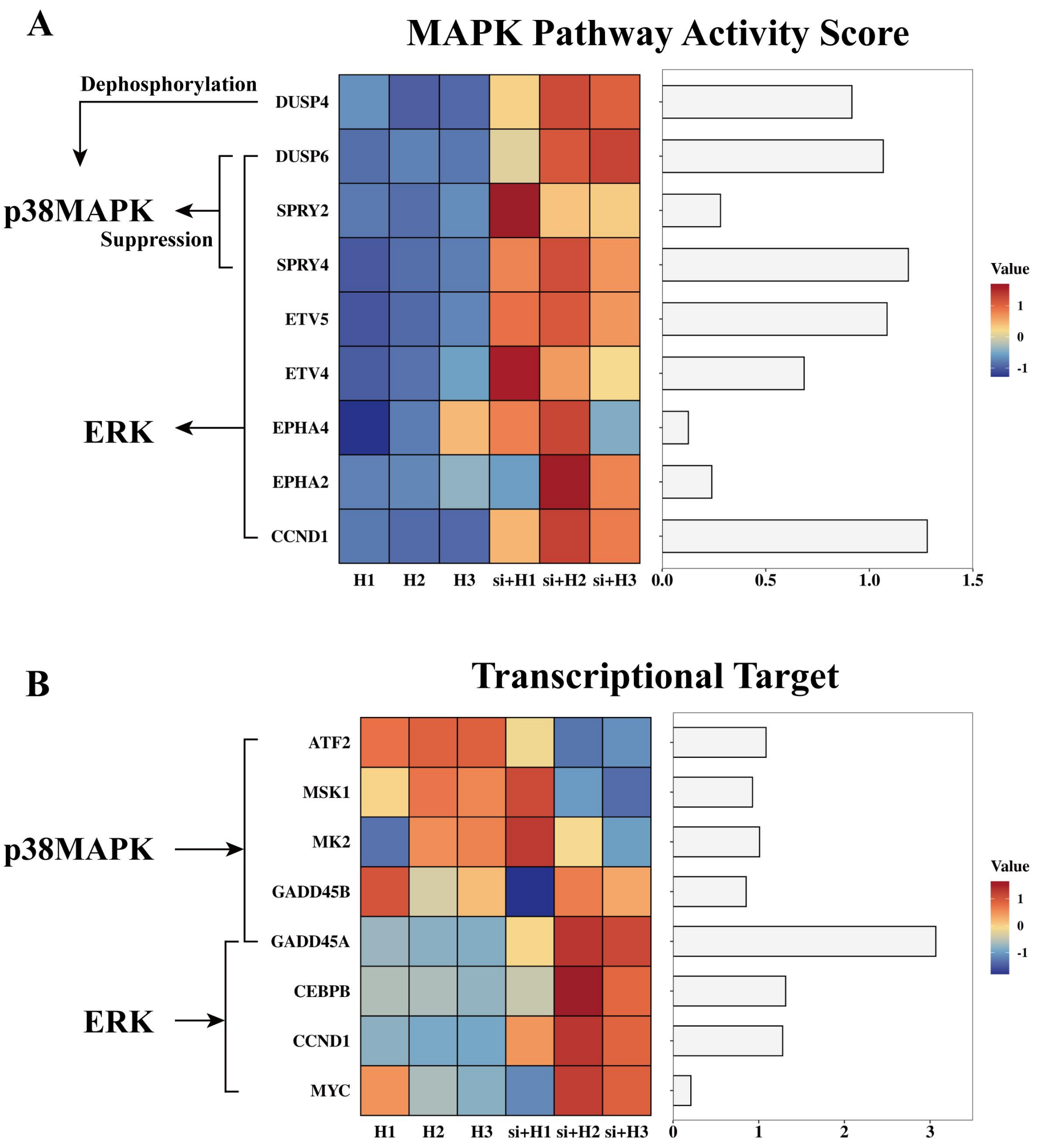

Supplemental Figure 4. The expression levels of MAPK pathway-related components in HBECs after BMAL1 knockdown.

(A) Heatmap demonstrated the expression of MAPK pathway activity score. **(B)** The expression of the P38MAPK pathway related and ERK pathway related genes from RNA-seq.

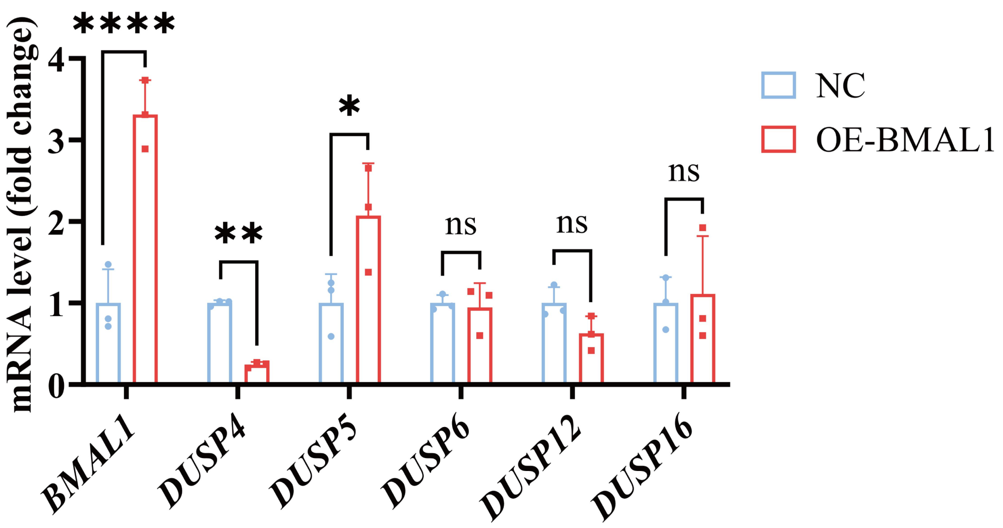

Supplemental Figure 5. mRNA levels of DUSPs in HBECs after BMAL1 overexpression.

The mRNA level of *BMAL1*, *DUSP4*, *DUSP5*, *DUSP6*, *DUSP12* and *DUSP16* in the HBECs.

**P*<0.05, ***P*<0.01, ****P*<0.001, *****P*<0.0001

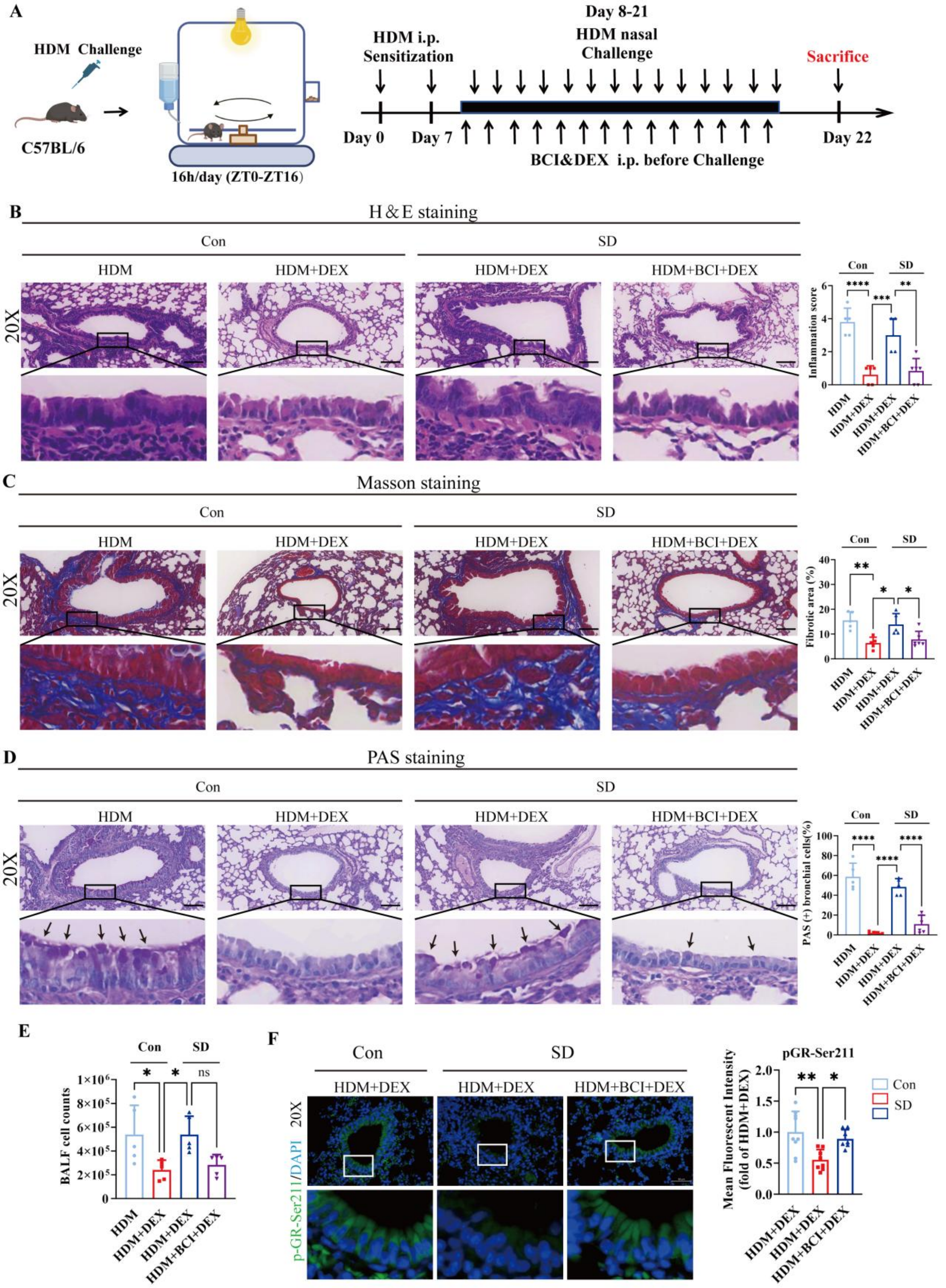

Supplemental Figure 6. Therapeutic effects of DEX and BCI in sleep deprivation mouse model of HDM-induced asthma.

(A) Schematic diagram of the SD mouse model with DEX and BCI treatment. **(B)** Representative images of H&E-stained lung tissue sections from the different groups

(scale bar =100 μm). The inflammation score was determined. **(C-D)** The amount of collagen around the airways and the percentages of PAS-positive airway epithelial cells were quantified (scale bar =100 μm). **(E)** Numbers of total cells in the BALF. **(F)** Immunofluorescence staining demonstrated the level of p-GR-Ser211 in airway epithelium (scale bar =50 μm).

**P*<0.05, ***P*<0.01, ****P*<0.001, *****P*<0.0001

A

B

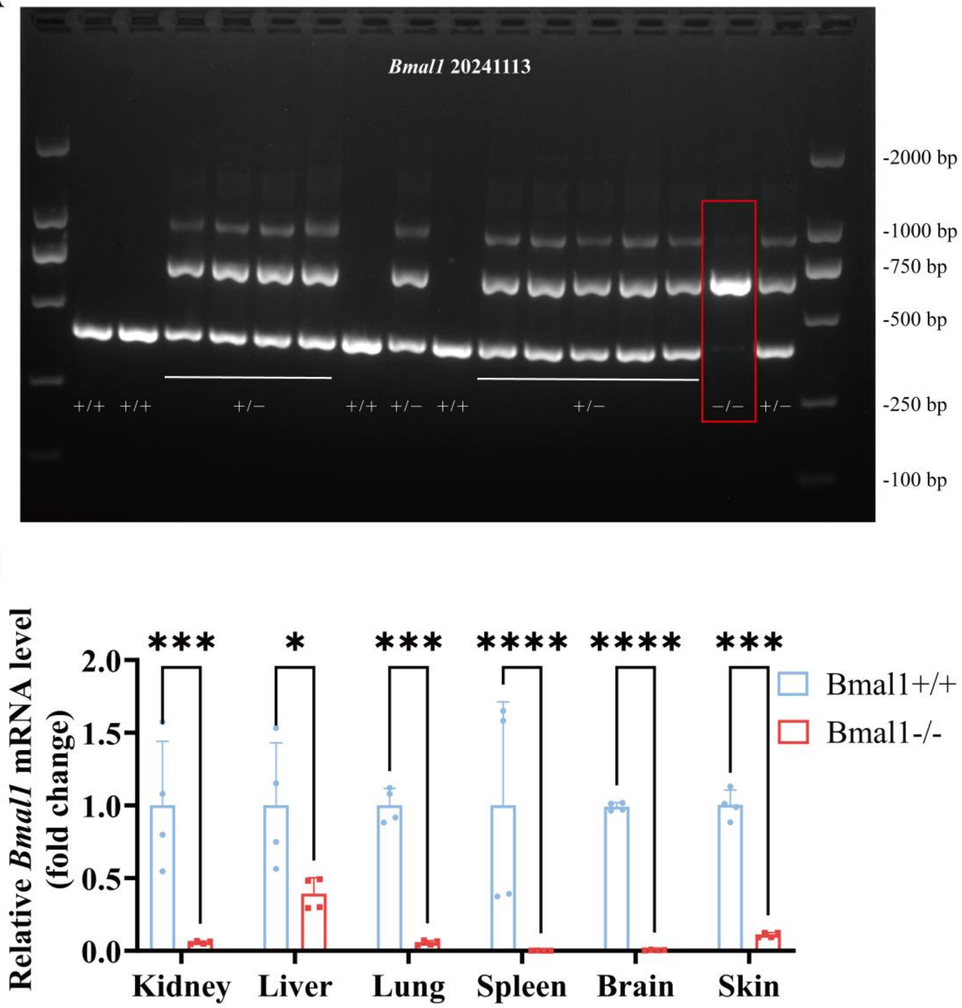

Supplemental Figure 7. Genetic identification of *Bmal1*$^{-/-}$ mice

(A) PCR genotyping of tail biopsies showing bands of 400 bp and 600 bp indicating the presence of the wild-type (only 400 bp), mutant (400bp and 600 bp) and knock out mice (only 600 bp). **(B)** mRNA expression of *Bmal1* in organs including kidney, liver, lung, spleen, brain and skin.

**P*<0.05, ***P*<0.01, ****P*<0.001, *****P*<0.0001
